# A Vascularized Microphysiological System Reproducing Endochondral Ossification in Vitro to Study Ewing Sarcoma Proliferation and Migration

**DOI:** 10.1002/adfm.202418470

**Published:** 2025-08-03

**Authors:** Maria Vittoria Colombo, Chiara Arrigoni, Tobias Faehling, Antonietta Verrillo, Viviana Secci, Thomas G. P. Grünewald, Giuseppe Talò, Alexandra Kummer, Jorge Gonzalez, Vincenzo De Rosa, Christian Candrian, Andrea Barbero, Florencia Cidre-Aranaz, Matteo Moretti

**Affiliations:** Regenerative Medicine Division, Institute for Translational Research, Ente Ospedaliero Cantonale - Università della Svizzera Italiana, via F Chiesa 5, Bellinzona 6500, Switzerland; Department of Chemistry, Materials and Chemical Engineering G.Natta, Politecnico di Milano, Piazzale Leonardo da Vinci 32, Milan 20100, Italy; Regenerative Medicine Division, Institute for Translational Research, Ente Ospedaliero Cantonale - Università della Svizzera Italiana, via F Chiesa 5, Bellinzona 6500, Switzerland; Euler Institute, Biomedical Sciences Faculty, Università della Svizzera Italiana (USI), via Buffi 13, Lugano 6900, Switzerland; Hopp Children’s Cancer Center Heidelberg (KiTZ), 69120 Heidelberg, Germany; Division of Translational Pediatric Sarcoma Research, German Cancer Research Center (DKFZ), German Cancer Consortium (DKTK), 69120 Heidelberg, Germany; National Center for Tumor Diseases (NCT), NCT Heidelberg, a partnership between DKFZ and Heidelberg University Hospital, 69120 Heidelberg, Germany; Regenerative Medicine Division, Institute for Translational Research, Ente Ospedaliero Cantonale - Università della Svizzera Italiana, via F Chiesa 5, Bellinzona 6500, Switzerland; Regenerative Medicine Division, Institute for Translational Research, Ente Ospedaliero Cantonale - Università della Svizzera Italiana, via F Chiesa 5, Bellinzona 6500, Switzerland; Department of Chemistry, Materials and Chemical Engineering G.Natta, Politecnico di Milano, Piazzale Leonardo da Vinci 32, Milan 20100, Italy; Euler Institute, Biomedical Sciences Faculty, Università della Svizzera Italiana (USI), via Buffi 13, Lugano 6900, Switzerland; Hopp Children’s Cancer Center Heidelberg (KiTZ), 69120 Heidelberg, Germany; Division of Translational Pediatric Sarcoma Research, German Cancer Research Center (DKFZ), German Cancer Consortium (DKTK), 69120 Heidelberg, Germany; Institute of Pathology, Heidelberg University Hospital, 69120 Heidelberg, Germany; National Center for Tumor Diseases (NCT), NCT Heidelberg, a partnership between DKFZ and Heidelberg University Hospital, 69120 Heidelberg, Germany; Cell and Tissue Engineering Laboratory, IRCCS Istituto Ortopedico Galeazzi, Via Cristina Belgioioso, 173, Milan 20161, Italy; Hopp Children’s Cancer Center Heidelberg (KiTZ), 69120 Heidelberg, Germany; Division of Translational Pediatric Sarcoma Research, German Cancer Research Center (DKFZ), German Cancer Consortium (DKTK), 69120 Heidelberg, Germany; National Center for Tumor Diseases (NCT), NCT Heidelberg, a partnership between DKFZ and Heidelberg University Hospital, 69120 Heidelberg, Germany; Pediatric Institute of Southern Switzerland, Ente Ospedaliero Cantonale, Bellinzona 6500, Switzerland; Pediatric Institute of Southern Switzerland, Ente Ospedaliero Cantonale, Bellinzona 6500, Switzerland; Euler Institute, Biomedical Sciences Faculty, Università della Svizzera Italiana (USI), via Buffi 13, Lugano 6900, Switzerland; Service of Orthopaedics and Traumatology, Department of Surgery, Ente Ospedaliero Cantonale, Via Tesserete 46, Lugano 6900, Switzerland; Department of Biomedicine, University Hospital Basel, University of Basel, Hebelstrasse, 20, Basel 4031, Switzerland; Hopp Children’s Cancer Center Heidelberg (KiTZ), 69120 Heidelberg, Germany; Division of Translational Pediatric Sarcoma Research, German Cancer Research Center (DKFZ), German Cancer Consortium (DKTK), 69120 Heidelberg, Germany; National Center for Tumor Diseases (NCT), NCT Heidelberg, a partnership between DKFZ and Heidelberg University Hospital, 69120 Heidelberg, Germany; Regenerative Medicine Division, Institute for Translational Research, Ente Ospedaliero Cantonale - Università della Svizzera Italiana, via F Chiesa 5, Bellinzona 6500, Switzerland; Euler Institute, Biomedical Sciences Faculty, Università della Svizzera Italiana (USI), via Buffi 13, Lugano 6900, Switzerland; Cell and Tissue Engineering Laboratory, IRCCS Istituto Ortopedico Galeazzi, Via Cristina Belgioioso, 173, Milan 20161, Italy

**Keywords:** endochondral ossification, ewing sarcoma, microphysiological system

## Abstract

Endochondral ossification (ECO) is the process at the basis of long bone development, occurring in children and involving vascularization and subsequent mineralization of a cartilage template. Deregulated bone developmental pathways could correlate with pediatric bone tumors, including Ewing Sarcoma (EwS), an aggressive tumor with a poor prognosis for patients with recurrent or metastatic disease. Considering the limitations of available EwS preclinical models, at developing a microphysiological system recapitulating bone developmental steps are aimed, to investigate their influence on EwS cell proliferation and migratory behavior. This system includes spheroids of mesenchymal stromal cells sequentially differentiated in chondrogenic and hypertrophic stages within a fibrin hydrogel, in the presence or not of vascular cells and mineralized particles. It shows that by modulating system components it is possible to recapitulate hypertrophic ECO stage, in a higher extent with the addition of vascular cells without a mineral component. Hypertrophic conditions foster the proliferation of EwS cells as compared to avascular and mineralized environments and support the migration of highly aggressive EWSR1::FLI1_low_ cell phenotype, contrarily to empty 3D fibrin gel. Overall, the results support the use of the ECO-mimicking microphysiological system as a valuable preclinical model to investigate the role of developmental mechanisms in EwS onset and progression.

## 1 Introduction

Bone longitudinal growth primarily occurs through a process called Endochondral ossification (ECO), starting during embryonic development and continuing until ≈25 years of age.^[1]^ ECO begins with the formation of a cartilage template that is gradually replaced by bone through several stages. Initially, mesenchymal stem/stromal cells (MSCs) condense to form cartilage tissue and differentiate into chondrocytes. During the next stage, chondrocytes undergo hypertrophy and produce a type X collagen-rich avascular cartilaginous matrix and deposit hydroxyapatite to this extracellular matrix. Hypertrophic chondrocytes release growth factors such as Vascular Endothelial Growth Factor (VEGF), promoting vessel invasion and attracting bone progenitor cells, which use the cartilage matrix as a template for bone matrix deposition, leading to the formation of two secondary centers of ossification.^[2]^ During puberty, these ossification centers fuse, and the growth plate disappears, resulting in bone maturation.^[3]^

Several efforts have been undertaken to engineer bone tissue constructs recapitulating this complex process, generally involving a first in vitro phase, in which MSCs are differentiated in chondrocytes and then induced toward a hypertrophic phenotype, followed by in-vivo implantation to achieve vascularization and mineralization.^[4–7]^ However, with this approach, chimeric constructs with blood vessels derived from mouse surrounded by human bone-like tissue were generated. As an alternative, tissue-engineered ECO models based on human cells and cultured only in vitro have been proposed, allowing to recapitulate the mineralization process in 3D aggregates of MSCs and vascular cells.^[8]^ Overall, existing models achieved the reproduction of some hallmarks of the ECO process, although a preclinical tool that can be used for the investigation of pathologies affecting the bone during its development has not been generated yet.

Among diseases arising in pediatric bones, one of the most devastating is Ewing Sarcoma (EwS), a bone tumor affecting children, adolescents and young adults with 80% of cases diagnosed in individuals under 18 years of age.^[9]^ Despite the current treatment regimen of multi-drug chemotherapy and local treatments such as surgery and radiotherapy, the prognosis remains poor for patients with recurrent or bone metastatic disease, with a survival rate lower than 20%. This is particularly concerning, since 25% of cases presents already metastatic disease at the time of initial diagnosis.^[10,11]^

EwS is genetically characterized by a specific chromosomal translocation that fuses a member of the FET gene family with a member of the ETS transcription factor family, resulting in ≈15 different fusion proteins in the EWS::ETS domain.^[12]^ The most common fusion gene is EWSR1::FLI1, which is present in 85% of EwS cases. The heterogeneity of EWSR1::FLI1 expression modulates EwS cell invasiveness, making this fusion protein a critical key for the initiation of the metastatic mechanism and an interesting target for novel therapies.^[13]^ Although EwS is genetically well characterized, its cell of origin remains a matter of debate, in which several hypotheses have been made, including the possibility that EwS arises from cellular progenitors in the developing bone microenvironment such as neural crest cells and mesenchymal stem cells.^[14]^ Additionally, the young age of incidence, both for primary and metastatic tumors, further suggests that deregulation of developmental pathways and microenvironmental factors specifically active during bone growth may play a role in the development of EwS and its progression to metastasis.

In this context, various transcription factors guide cell differentiation during the ECO process and these can possibly foster EwS tumor onset and progression. In particular, we have previously shown that the oncoprotein EWSR1::FLI1 can bind to a region within the SOX6 gene (a key transcription factor involved in ECO), inducing its high and constitutive overexpression in EwS cells, hence promoting their proliferation and tumorigenicity.^[15]^ Conversely, a different study indicated that knockout of EWS gene in zebrafish directly regulated SOX9 expression, leading to impaired hypertrophic differentiation of cartilage, thus suggesting a role of EWS pathway in skeletal development.^[16]^ These findings provide a basis for future studies on the crosstalk between the processes of EwS onset and metastasis formation and bone development.

The investigation of this crosstalk however requires preclinical tumor models that can recreate the physiological microenvironment of the growing bone. Despite multiple efforts, appropriate EwS modelling is still lacking, primarily because all attempts to develop transgenic mouse models recapitulating human tumor progression have not been successful yet. ^[17]^ Furthermore, in vitro models such as tumor spheroids are limited in replicating the complexity of the tumor microenvironment and lack the adequate spatial architecture to mimic the process of cell migration and metastasis. To overcome these issues, tissue engineered EwS models have been described, incorporating components of the tumor microenvironment within scaffolds (i.e., hydrogels, 3D printed or electrospun scaffolds, decellularized scaffolds) or using microfluidic techniques enriched with ECM components, such as mineralization by osteogenic MSCs,^[18]^ or seeded with bone derived cells, as osteoblasts and osteoclasts.^[19,20]^ However, all these models lack the vascular component, which has a key role in the metastatic process^[21]^ and they replicate the mature bone, whereby the process of ossification is completed and the ECM is fully mineralized. Overall, the development of an adequate EwS model that more accurately reflect the complexity and heterogeneity of this disease could allow the researchers to identify new therapeutic targets and test new treatments. To this end, in this study we describe a new human microphysiological system for the investigation of the crosstalk between EwS and an ECO environment. Our approach implements a complex spatial architecture, exploiting multicellular spheroid cultures combined with 3D printing technologies, to recapitulate the subsequent ECO steps while monitoring the migration and clustering of EwS cells in response to an ECO microenvironment. In particular, we embedded hypertrophic cartilagelike spheroids in a fibrin gel encased in a custom designed 3D printed support, with or without the presence of invading vascular cells and a mineral component. By leveraging the possibility to dissect multiple steps of the ECO process, our model offers a unique opportunity to better understand the crosstalk between EwS tumor and its evolving microenvironment, possibly highlighting new targets to hinder its growth and metastatic diffusion.

## 2 Results

### 2.1 Characterization of Chondrogenic and Hyperthrophic Spheroids

During the first ECO phase, MSCs must condense to form a cartilaginous matrix. This process can be achieved by culturing bone marrow-derived MSCs (BMSCs) in micro-aggregates (spheroids, SPH) to induce cell-to-cell interactions and mimic the condensation that occurs during the early stages of ECO.^[22]^ Once condensation is achieved, chondrogenic priming of BMSCs is induced by culturing them in specific medium, known to promote chondrogenesis.^[6]^ In this study, we used BMSCs derived from pediatric healthy donors and firstly confirmed their mesenchymal lineage and ability to differentiate toward the osteo-chondrogenic phenotype. BMSCs cultured as monolayer expressed CD73, a surface marker specific for MSCs, and had a typical fusiform shape (Figure S1a, Supporting Information).^[23]^

**Figure 1:**
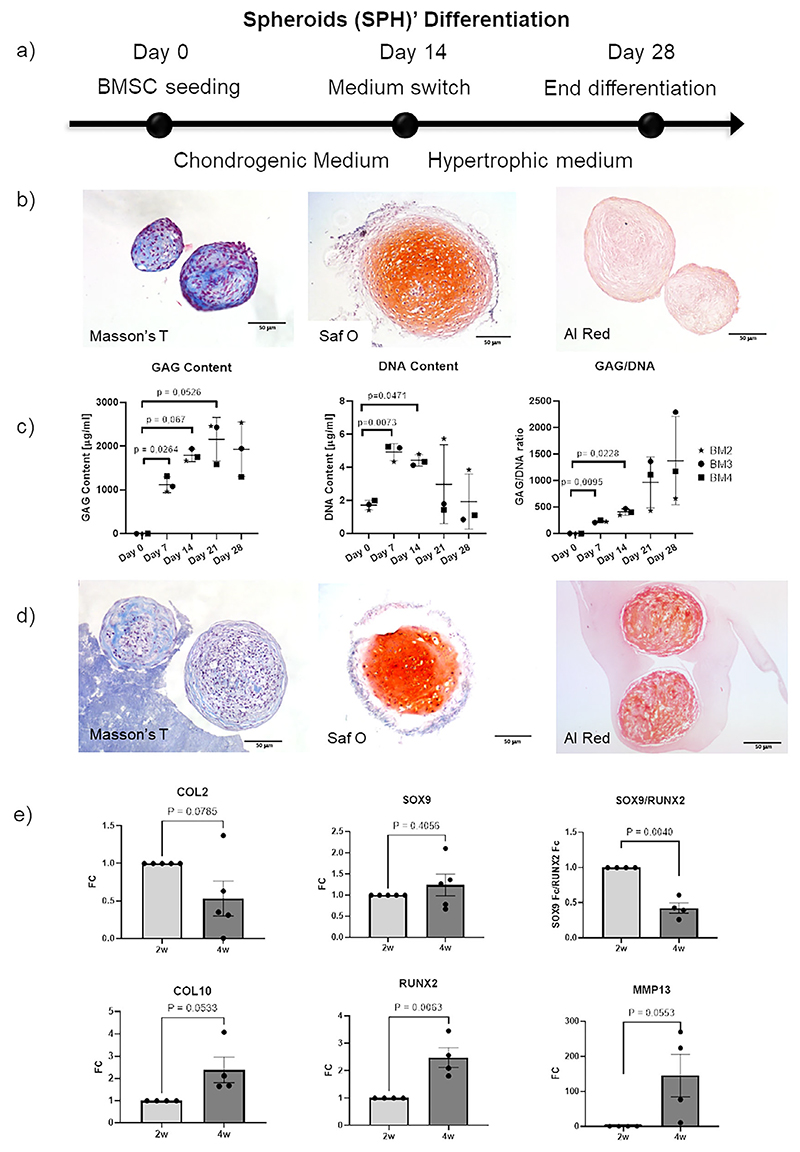
(Characterization of SPH differentiation. a) SPHs differentiation schematic. Undifferentiated BMSCs were seeded in 96 well plates at day 0 to allow cell aggregation. After 14 days of chondrogenic differentiation, the media was switched to a hypertrophic one to reach hypertrophic differentiation of aggregates. At day 28, SPHs were used for characterization or seeded in the device. b,d) Histological staining of differentiating SPHs. SPHs were stained at day 14 b), after chondrogenic differentiation, and at day 28 d), after hypertrophic differentiation. First column shows the results of Masson’s trichrome staining: nuclei are stained in pink, collagen is stained in blue. Collagen matrix decreases after hypertrophic differentiation (day 28). Second column shows the Safranin O (SafO) proteoglycan staining with a Fast green counter staining. At day 14 the proteoglycan content is homogeneous in the SPH, whilst at day 28 is lower and non-uniformly distributed. Third column shows the results of Alizarin red (Al Red) staining for calcium deposition, which is higher at day 28. c) GAG and DNA evaluation during differentiation of SPHs (*n* = 3 donors). P value determined with Tukey’s multiple comparisons test. All graphs show individual values of each BMSCs donor. e) Chondrogenic (top row) and hypertrophic (bottom row) gene expression (*n* = 4–5). ΔCt was normalized over RPLP0. The fold change was calculated as differentiated SPHs at day 28 over SPHs at day 14. P values calculated with unpaired t test).

To assess their osteogenic potential, BMSCs were differentiated in 2D conditions in osteogenic medium for 10 days. After culture they showed high expression of collagen 1 (*COL1*), osteopontin (*OPN*) and osteocalcin (*OCL*) (Figure S1b, Supporting Information), indicating their capability to differentiate toward the osteogenic lineage. To further confirm osteogenic differentiation, we analyzed the expression of genes typically enriched in osteoblasts including collagen type 1, alpha 1 (*COL1A1), Runt-related transcription factor 2 (RUNX2)*, and bone gammacarboxyglutamic acid-containing protein (*BGLAP)*, and found an increased expression as compared to undifferentiated cells, even if in a different extent within different donors. In particular, BM2, BM3, and BM4 presented a higher expression level of osteogenic genes, including *COL1A1, RUNX2* and *BGLAP* (i.e., osteocalcin) as compared to BM1 (Figure S1c, Supporting Information). Then, to verify the possibility to achieve chondrogenic SPHs, we seeded BMSCs in low-attachment 96-well plate and left them in 3D culture for 14 days, where they aggregated in spheroids of 957.6 ± 23 μm average diameter. Safranin-O staining at 14 days showed chondrogenic differentiation of SPHs (Figure 1b) to varying degrees, depending on the donor (Figure S1d, Supporting Information).

In agreement with our previous results, BM2, BM3, and BM4 exhibited intense Safranin O staining and collagen 2 expression, while BM1 showed lower levels of collagen and matrix deposition. To confirm chondrogenic differentiation, we measured glicosamminoglycans (GAG) content, which increased constantly from day 1 to day 14 (Figure 1c), although there were clear differences among the donors. In particular, BM1 showed no release of GAG with almost negligible GAG/DNA ratio. Due to these results and the low capability of chondrogenic differentiation, BM1 was excluded from further experiments. Finally, DNA content increased in all SPHs from days 0 to 7, and remained unchanged at day 14 (Figure 1c), further supporting the presence of a proliferative phase of the cells during the initial condensationchondrogenic stage, as expected.

After the induction of a chondrogenic differentiation, ECO progresses through a hypertrophy stage, thus we cultured our chondrogenic SPHs in hypertrophic medium for further 14 days. At the end of hypertrophic culture, SPHs showed stable content of GAGs and a lower content of collagen, as suggested by the less intense staining observed with Masson’s trichrome at day 28 as compared to day 14. Furthermore, SPHs after hypertrophic differentiation showed calcium deposition, as indicated by intense Alizarin red staining, which was not present in SPHs at day 14 (Figure 1d). This was further supported by the GAG quantification, which showed a plateau after 14 days, suggesting that the deposition of cartilage matrix remained stable during hypertrophic differentiation. Regarding cell proliferation, from days 14 to 28 DNA production remained steady (Figure 1c), similarly to what happens during the ECO process, in which, after an initial proliferating phase, chondrocytes stop to proliferate and initiate to enlarge and deposit ECM.^[3,8]^ To confirm the subsequential differentiation stages of SPHs, chondrogenic and hypertrophic markers were analyzed by RT-qPCR. Collagen, type II, alpha 1*(COL2A1)* gene expression was detected after 14 days of chondrogenic differentiation, and decreased at day 28. *COL2* is a key marker of chondrogenic phenotype and is the major component of the cartilage ECM released by the chondrocytes.^[3]^ In addition, the expression of *SOX9*, a master transcription factor of chondrogenic development,^[24]^ was high in the 14-day SPHs and remained stable at day 28 (Figure 1e). Looking at the expression of hypertrophic genes, at day 28 collagen type 10 (*COL10*), a hallmark of hypertrophic phenotype,^[25]^ showed an almost significantly higher expression compared to day 14, suggesting that a two-week differentiation period of SPH in 96-well plates increased its expression but may not be sufficient to reach a fully hypertrophic stage. Notwithstanding, the expression of other key hypertrophic markers such as *RUNX2* and matrix metallopeptidase 13 (*MMP13)* showed a significant or almost significant increase, consistent with the progression of the ECO process. *MMP13* is known to be involved in collagen degradation in the extracellular matrix, fundamental for facilitating vascularization^[26]^ whilst *RUNX2* plays a key role in promoting hypertrophic differentiation,^[27]^ and its expression increased from days 14 to 28.

Overall, our results confirmed that our pediatric BMSCs were capable of differentiating toward both chondrogenic and osteogenic lineages, and can be induced toward a hypertrophic differentiation, although at different levels, further supporting evidences on the variability in the differentiation of BMSCs isolated from different subjects.^[28]^

### 2.2 Development of the 3D Printed Device

**Figure 2:**
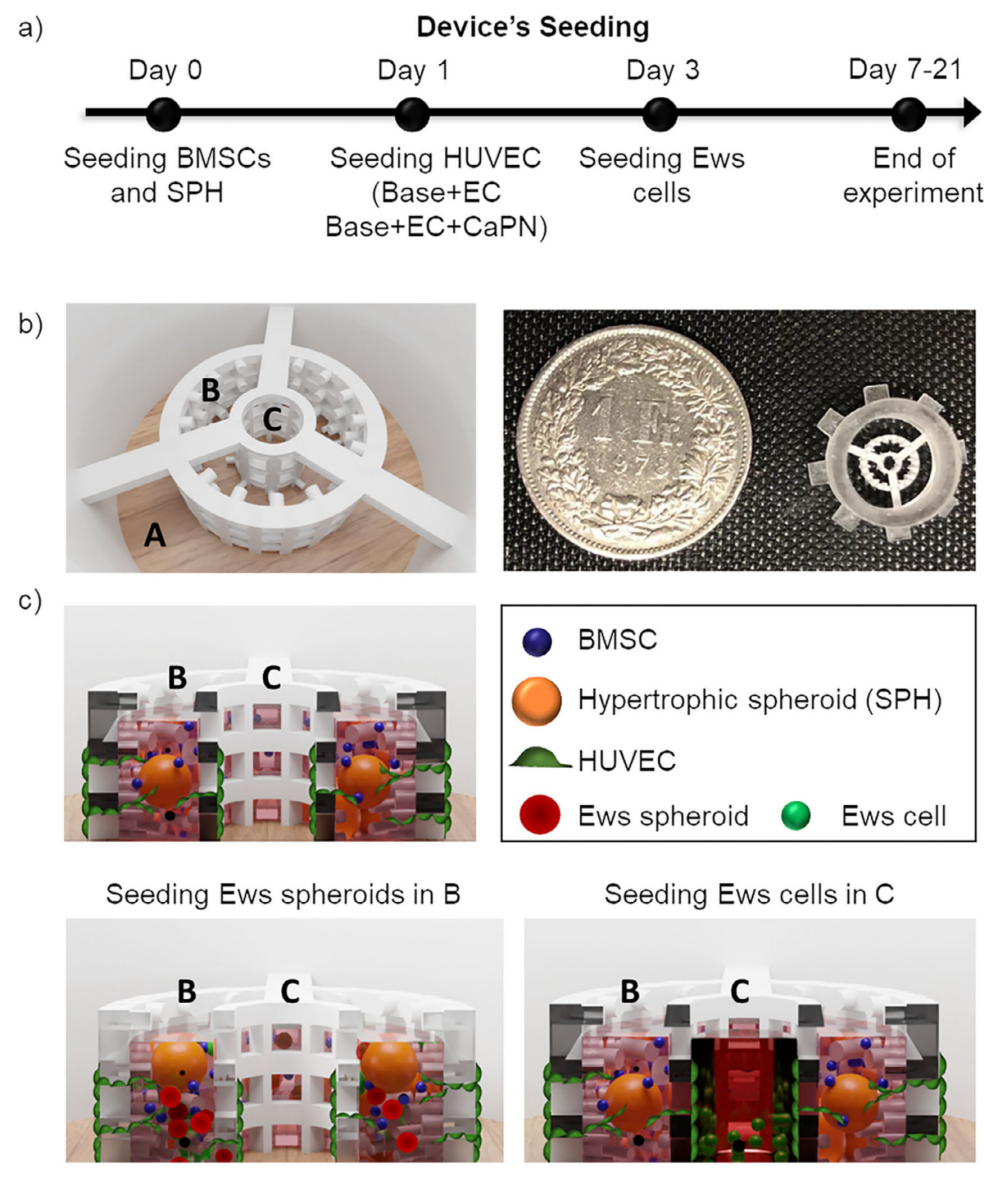
(Schematics of experimental procedures and device renderings. a) Device seeding and culture procedure: after SPHs differentiation, they were seeded into the central chamber (B) of the devices embedded in a fibrin gel with BMSCs and Calcium Phosphate Nanoparticles (CaPN), where required. For tumor cell proliferation experiments, EwS cells were embedded as single cells in the same gel. The day after, devices with vascularization were seeded with endothelial cells (EC: HUVECs), and then left in culture for 7 days. For tumor cell migration experiments, EwS cells were seeded after 3 days in the innermost chamber (C) of the device and kept in culture up to 21 days. b) Top view rendering (left) and photograph (right) of the empty 3D printed device showing the three concentrical chambers divided by two open-pores grids. The external one (A) has a higher border to contain the culture medium, the internal ones are communicating to allow cell migration and nutrient exchange. c) Cross-section rendering of the middle and inner chamber, showing the position of SPH, BMSCs, HUVECs and EwS cells in the different experimental conditions.

The 3D printed device design included different chambers to guarantee the compartmentalization of different microenvironments (Figure 2b) and to allow for testing migration and proliferation of tumor cells. In particular, it consists of three concentrical and communicating chambers divided by two grids with open pores. The outer chamber (A) is dedicated to culture medium collection, whilst the other two chambers (B and C) are dedicated to cell culture, specifically B hosts the gel with SPH, whilst C is for cancer cell seeding (Figure 2c).

**Figure 3:**
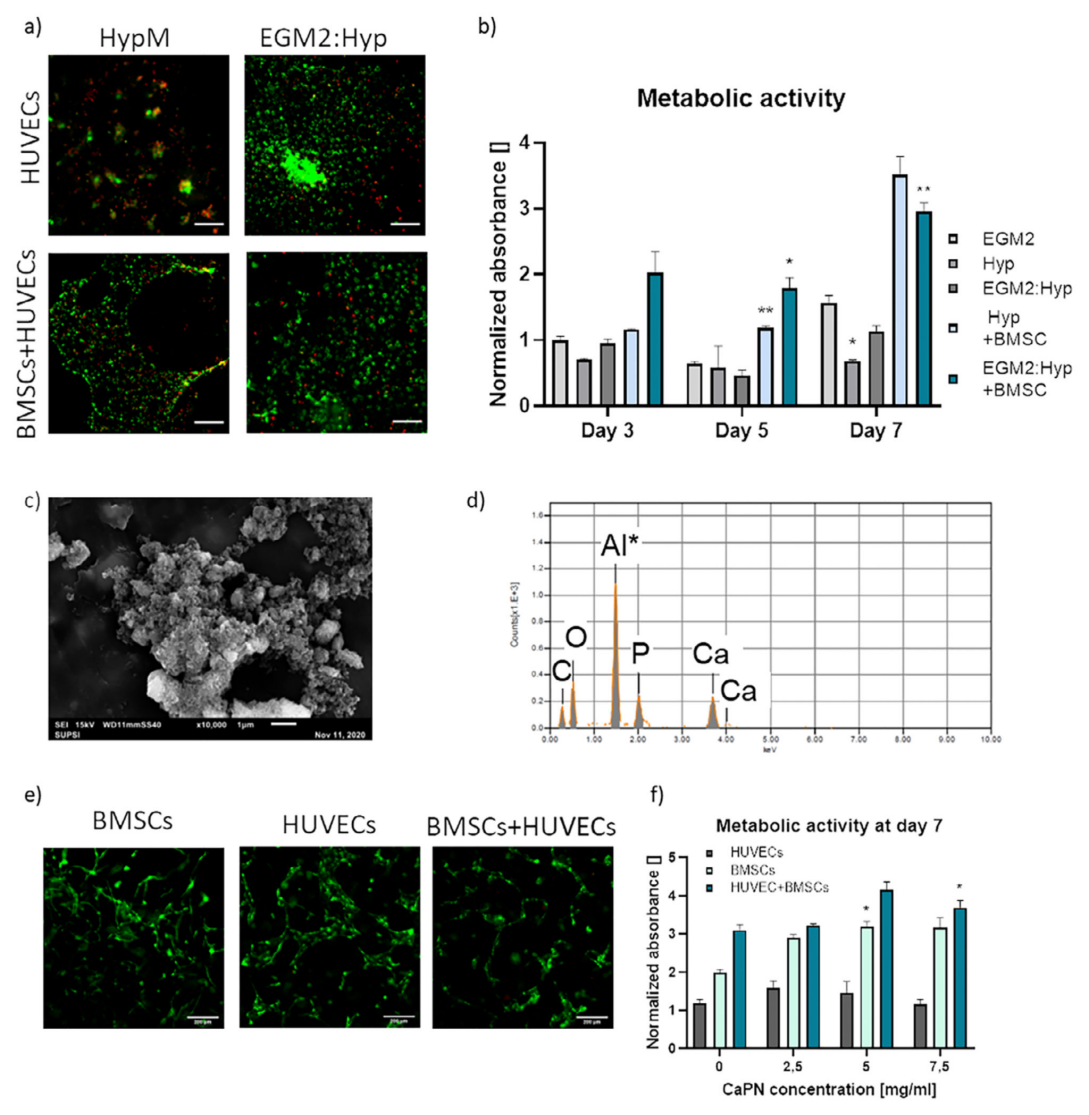
(Setup of the ECO model. a,b) Effect of different media composition on cell viability a) Fluorescence images of live (green) and dead (red) cells (HUVEC w or w/o BMSCs) after 7 days of culture. Scale bar = 200 μm. b) Normalized absorbance values obtained from Alamar Blue assay after 3, 5, and 7 days of culture. Values are reported as average and standard error (*n* = 3). P value determined with Dunnett’s multiple comparisons test versus control condition EGM2. ^*^p < 0.05; ^**^ *p* < 0.01. c–f) Characterization and effect of calcium nanoparticles on cell viability. c) Scanning Electron Microscopy (SEM) image showing the CaPN morphology and micrometric aggregation. Scale bar = 1 μm. d) Energy dispersive X-ray (EDX) spectroscopy showing the presence of phosphate and calcium in the particles. Al is the signal of the SEM plate. e) Live and dead staining of cells embedded in fibrin drops at day 7. Only calcein (green) staining is shown due to red autofluorescence of CaPN. Scale bar = 200 μm. f) Normalized absorbance values obtained from Alamar Blue assay with different CaPN concentrations at day 7. Values are reported as average and standard error (*n* = 3). P values measured with Dunnett’s multiple comparisons test on 0 mg mL^−1^ for each cell type, ^*^p < 0.05).

The ECO process involves different types of cells and matrices. For our model, we included vascular cells (HUVECs), BMSCs and hypertrophic SPHs, requiring different media and specific cell densities. To first identify the best coculture medium, three media compositions were selected: EGM2, Hyp medium (HypM) and a mix 1:1 of EGM2 and HypM. We measured the viability (images in Figure 3a) and metabolic activity (graph in Figure 3b) of HUVECs, alone or in coculture with BMSCs (from 3 donors) in the device, showing that at 7 days the combination EGM2:HypM offered a significantly higher viability of cells, in the presence of BMSCs.Therefore, this medium was chosen for co-culture experiments.

Further, to mimic the mineral matrix of bone, Calcium Phosphate Nanoparticles (CaPN) were introduced in the fibrin gel, aiming at promoting a more advanced stage of ECO.^[3]^ CaPN enriched fibrin offers a stiffer gel compared to the plain one and it could give a pre-mineralized matrix with physical and mechanical properties more similar to native bone.^[29]^ Nanoparticles were synthetized and analyzed through SEM and EDX to assess their composition and dimensions (Figure 3c,d). CaPN formed aggregates with variable dimensions, ranging between 0.114 and 2.4 μm. EDX analysis confirmed the presence of Ca (14.26% of mass) and P (7.9% of mass), in a ratio compatible with that of hydroxyapatite (Ca_10_(PO_4_)_6_(OH)_2_). Then, to evaluate the cytotoxicity of CaPN, fibrin drops with embedded BMSCs and/or HUVECs and different concentrations of nanoparticles (i.e., 0, 2.5, 5, and 7.5 mg mL^−1^) were seeded in 96-well plates. After seven days of culture, no significant difference in viability was detected between cells exposed at different concentrations of CaPN, indicating that even high concentrations of CaPN are not cytotoxic for cells (Figure 3e,f). Therefore, for subsequent seeding in the device, 7.5 mg mL^−1^ of CaPN in 10 mg ml^−1^ fibrin gel were used, a value still below the physiological amount of CaP that can be found in a native bone matrix (accounting for up to the 65% of bone weight)^[30]^ further recapitulating a growing bone microenvironment where complete mineralization has not yet been reached. Summarizing, the preliminary experiments allowed to optimize the parameters of our 3D model which maximize cell viability (7.5 mg mL^−1^ CaPN, EGM2+HypM (1:1)) which have been then used in following experiments.

### 2.3 Reaching Hypertrophy and Vascularization in 3D Co-Culture

**Figure 4:**
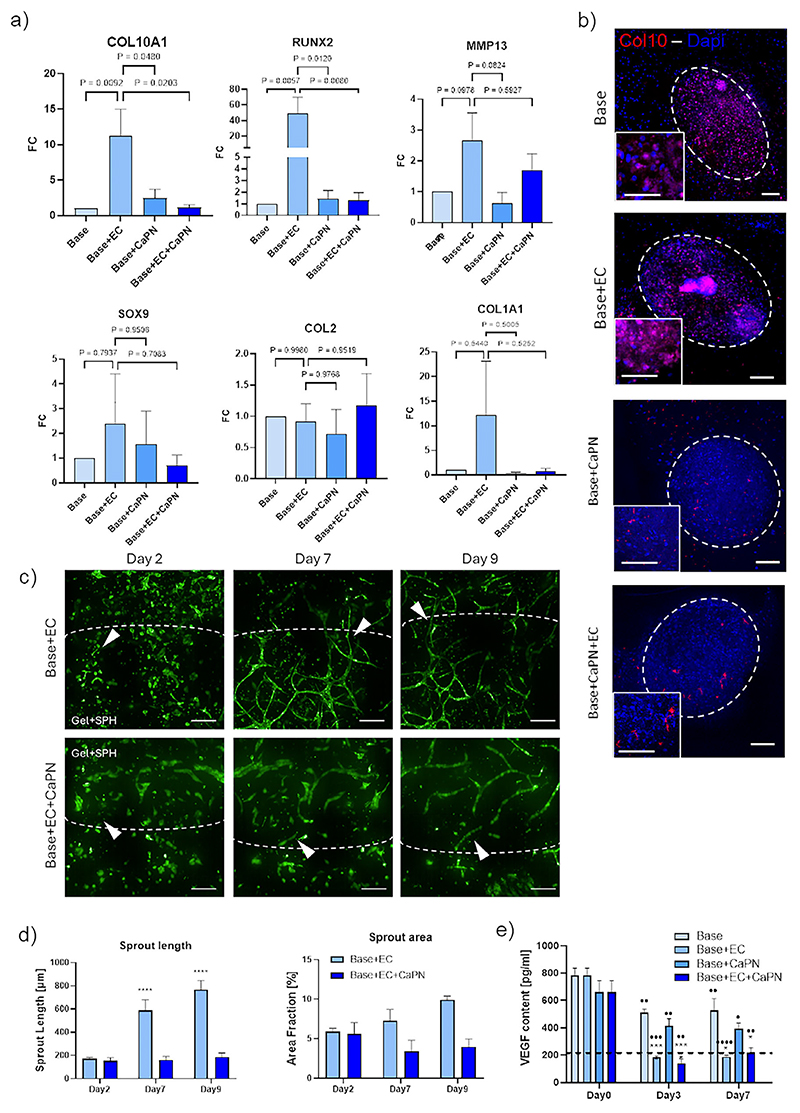
(ECO recapitulation in the device. a) Gene expression in the device after 7 days of co-culture in different compositions (Base, Base+EC, Base+CaPN, Base+EC+CaPN) for four BMSC donors. ΔCt was normalized over RPLP0. ΔΔCt was normalized on Base gene expression at day 7. b) Immunofluorescent staining of SPHs for collagen X in Base, Base+EC, Base+CaPN and Base+EC+CaPN conditions after 7 days. Col10 is present in the core of the aggregate, in a higher quantity in Base+EC conditions. Scale bars = 50 μm. c) Device vascularization. The HUVECs (white arrowhead) migrated from the external side of the grid into the middle chamber forming vascular networks up to day 9 (scale bar = 200 μm). d) First graph: length of vascular network in the middle chamber, in Base+EC and Base+EC+CaPN conditions. P values were calculated with two-way ANOVA. Day 2 versus Day 7 and Day 2 versus Day 9: ^****^, *p* < 0.0001; *N* = 8. Second graph: area covered by vessels in the middle chamber measured in 3 ROIs for *n* = 3 samples per condition. e) VEGF content in devices. Four different compositions of the devices and three time points (i.e., day 0, a pool of days 1, 2, and 3 (day3) and a pool of day 4,5,6, and 7 (day7) were considered (*n* = 6 for each condition). P values were calculated with 2way ANOVA. * compared to Base condition. ● compared to day 0. ^*^ *p* = 0.005, ^****^ *p* < 0.0001; ● *p* < 0.05, ● ● *p* < 0.01, ● ●● *p* < 0.001, ● ● ●● *p* = 0.0001; *N* = 6). The dashed line represents the content of VEGF measured in pure culture medium.

To mimic different stages of ECO, 3D-printed devices were seeded with four different cellular and matrix combinations: a base model with a co-culture of SPHs + BMSCs in 10 mg ml^−1^ fibrin gel, a base model enriched with 7.5 mg mL^−1^ CaPN (Base+CaPN), or with endothelial cells (Base+EC), and a base model enriched with both CaPN and endothelial cells (Base+EC+CaPN). After 7 days of co-culture, we analyzed the maturation stage of the ECO process by measuring the expression of ECO hallmark genes (Figure 4a). The presence of CaPN (Base+CaPN) did not seem to promote the expression of hypertrophic genes or the downregulation of chondrogenic ones as compared to Base conditions.

Interestingly, the combination inducing the highest expression of hypertrophic markers (*COL10, MMP13*, and *RUNX2*) was Base+EC, suggesting that the presence of endothelial cells promotes hypertrophic differentiation of the construct, in a higher extent as compared to the presence of a mineral component. This was further supported by the increased MMP13 expression in Base+EC+CaPN condition as opposed to Base+CaPN. Furthermore, the expression of chondrogenic genes such as COL2 and SOX9 was not different between the conditions with CaPN and/or ECs, whilst the expression of ECM genes such as COL1 was higher in the presence of ECs. Those results were confirmed by immunofluorescence, showing that Col2 was still expressed in SPHs, mainly localized near the nucleus and higher in the Base condition (Figure S2a, Supporting Information higher magnification insets). On the other hand, Col10, which was not significantly expressed in the hypertrophic SPHs, was found after 7 days in the core of co-cultured SPHs, in the Base and Base+EC conditions, whilst its presence was negligible in Base+CaPN and Base+EC+CaPN, suggesting the progression of hypertrophy in the complete device when vascularization was present but not with the addition of mineral particles (Figure 4b). The simultaneous presence of cartilaginous and hypertrophic extracellular matrix proteins is consistent with the structure of physiological growing bone, whereby areas at diverse differentiation degrees are present, with the proliferative and hypertrophic phenotypes coexisting.^[31]^ To mimic the vascularization of the hypertrophic construct, endothelial cells were added in the gel and seeded on the outer rims of the middle chamber as a monolayer at a concentration of 2*10^6^ cell/ml at day 1 (Figure 4c). After 7 days of culture in the model, HUVECs were able to invade the gel forming a vascular structure only in the Base+EC condition, whilst in presence of CaPN nanoparticles, no significant vessel formation was measured. At day 9, the vessels increased their area and length in Base+EC condition, with a slight increase also in Base+EC+CaPN (Figure 4d). To investigate if this vascular formation could be possibly driven by the production of VEGF by BMSCs and SPHs embedded in the gel, we measured the release of VEGF during culture in the four experimental conditions (Figure 4e). At day 0, all conditions were similar and close to values of control samples (i.e., SPHs after 28 days of differentiation) ranging from 661 to 783 pg mL^−1^. After three days of culture, the production of VEGF slightly decreased in all the conditions, with a lower value of VEGF content for EC containing samples. The same trend was observed at the following time point (Day7) in which the presence of VEGF was similar to Day3 and was significantly lower for Base+EC and Base+EC+CaPN compared to Base and Base+CaPN conditions. This lower VEGF content can be attributed to the expression of VEGF decoy receptor FMS-like tyrosine kinase 1 (FLT-1) by HUVECs, as shown in Figure S2b (Supporting Information). During ECO, the release of VEGF is necessary to induce the vascular invasion of the cartilage template,^[33,34]^ similarly, the increased production of VEGF by SPHs and BMSCs (as measured in Base and Base+CaPN models) could have stimulated the observed invasion of HUVECs from the monolayer into the early hypertrophic cartilage-like microenvironment.

### 2.4 Proliferation of EwS Cells into the ECO Model

**Figure 5:**
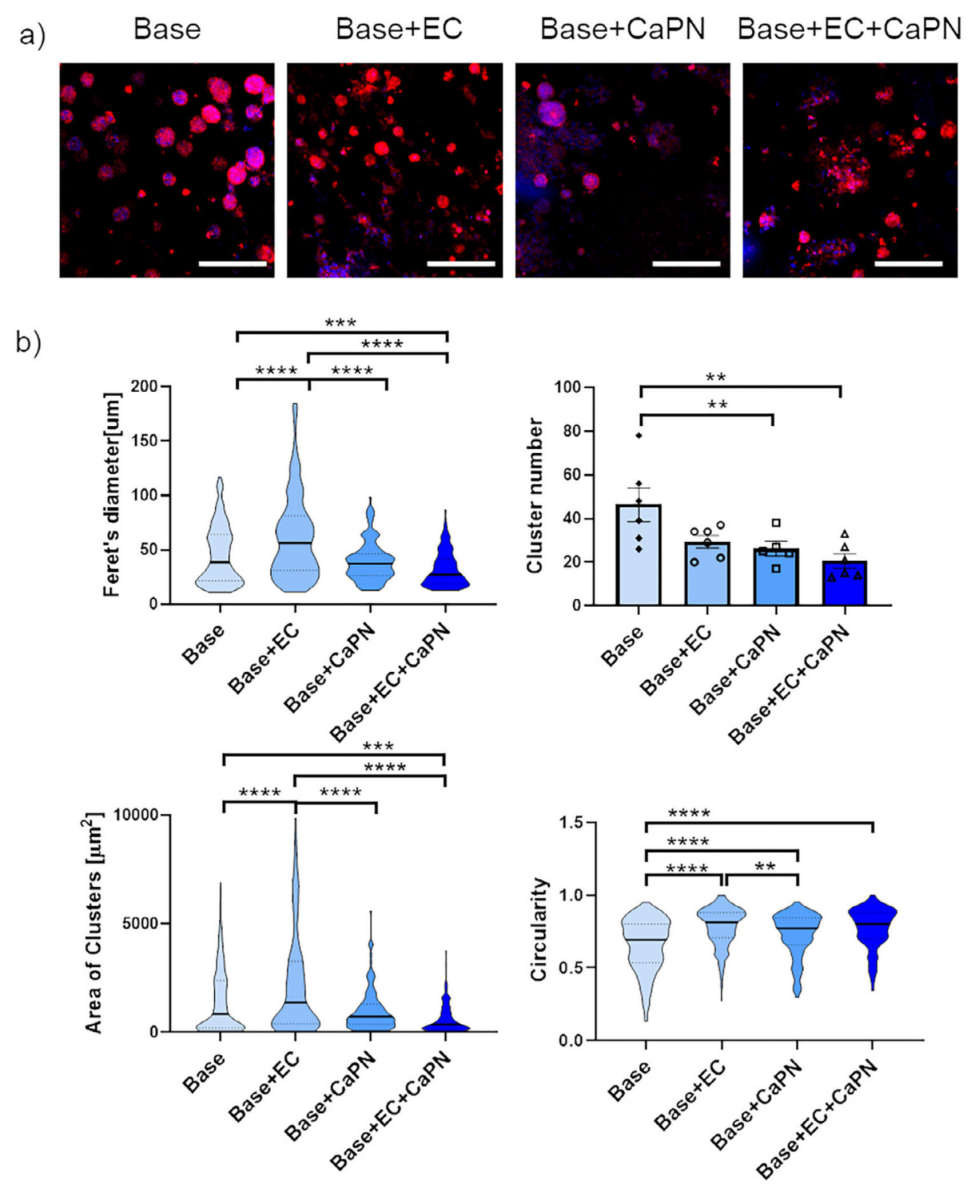
(EwS cell proliferation in ECO microenvironment. a) Immunofluorescence images of TC-32 clusters (red fluorescence, nuclei in blue) after 7 days of co-culture in the middle chamber. Cells developed clusters of different size and number based on the composition of the co-culture. *N* = 3 BMSC donors. Scale bar = 200 μm. b) Quantification of TC-32 cultured in devices in presence of SPHs. P values calculated with one-way ANOVA and Tukey’s multiple comparison test (*n* = 6 samples per condition). First graph represents the diameter of each cluster: ^***^, *p* = 0.0002; ^****^, *p* < 0.0001. Second graph represents the number of clusters: ^*^, *p* = 0.0472, ^**^, *p* = 0.0058. Third graph represents the area of each cluster: ^****^, *p* < 0.0001. Fourth graph represent the circularity of clusters: ^**^, *p* = 0.0027; ^****^, *p* < 0.0001).

After ECO model development and characterization, we investigated if different ECO microenvironments influenced the proliferation of EwS cells. After 7 days of culture, we compared a more hypertrophic cartilage-like model, i.e., Base, including only differentiated SPHs, with increasingly mature bone models, including also vasculature (i.e., Base+EC), mineralized component (i.e., Base+CaPN) or both (i.e., Base+EC+CaPN). In all the conditions, single TC-32 cells seeded were able to proliferate and form typical round, compact clusters in 7 days (Figure 5a). Quantification of the red fluorescent area in the different conditions showed a significantly higher area of EwS clusters when in co-culture with the Base+EC model, as compared to the other conditions (Figure 5 a and b). Interestingly, the Base+EC+CaPN condition showed significantly less and smaller TC-32 clusters as compared to the conditions w/o CaPN, suggesting that the presence of a mineral component may partly inhibit EwS cell proliferation. Furthermore, the cluster’s morphology was significantly more circular in the Base +EC, as compared to the other conditions (Figure 5b, bottom left).

To exclude the possibility that cluster growth could be limited by the steric hindrance of the SPHs, we seeded control conditions containing exclusively the cells in the fibrin gel (i.e., BMSCs only as in the Base model, BMSCs+ HUVEC as in the Base+EC, BMSCs+CaPN as in Base+CaPN and BMSCs with both HUVEC and CaPN as in Base+EC+CaPN). The Base model presented the same surface area of EwS cells as compared to the relative control condition, thus suggesting that the presence of SPHs per se did not influence the cluster growth (Figure S3, Supporting Information).

Surprisingly, in the Base+EC condition, EwS clusters were significantly bigger as compared to the relative control condition, highlighting that the presence of hypertrophic SPHs in a vascularized environment could foster EwS growth better than a simpler model (Figure S3b, Supporting Information). Conversely, EwS cell growth was significantly decreased in the models with SPHs and CaPN as compared to relative controls (Figure S3b, Supporting Information). The significant and opposite differences in EwS cell growth between conditions including SPHs and conditions with single cells dispersed in fibrin gel, suggest that the presence of hypertrophic aggregates, producing ECM molecules and possibly other secreted factors, can greatly influence tumor cell growth, beyond the effects of single cells embedded in the microenvironment, highlighting the need to wholly reproduce the complex ECO environment.

### 2.5 Cell Migration in the ECO Model

For a proof-of-concept investigation of the effects of the Base+EC microenvironment on the migratory behavior of EwS cells, we used EwS cell lines harboring a shRNA-mediated EWSR1::FLI1 fusion knockdown. The conditional fusion expression has been shown to shift the EwS cell phenotype from a proliferative one in the absence of Dox (EWSR1::FLI1_high_) to a more migratory one in the presence of Dox (EWSR1::FLI1_low_).^[13]^ We first performed a preliminary scratch test to verify the differences in cell migration with and w/o Dox-induced EWSR1::FLI1 KD and observed a faster scratch closure, indicating increased cell migration, in presence of Dox (Figure S4, Supporting Information).

We then verified that our device allowed monitoring migration of cancer cells into a fibrin-based matrix, by seeding highly metastatic SUM159 breast cancer cells in chamber C, and observed an evident cell migration toward the middle chamber (B) after 7 days (Figure S5, Supporting Information).

**Figure 6:**
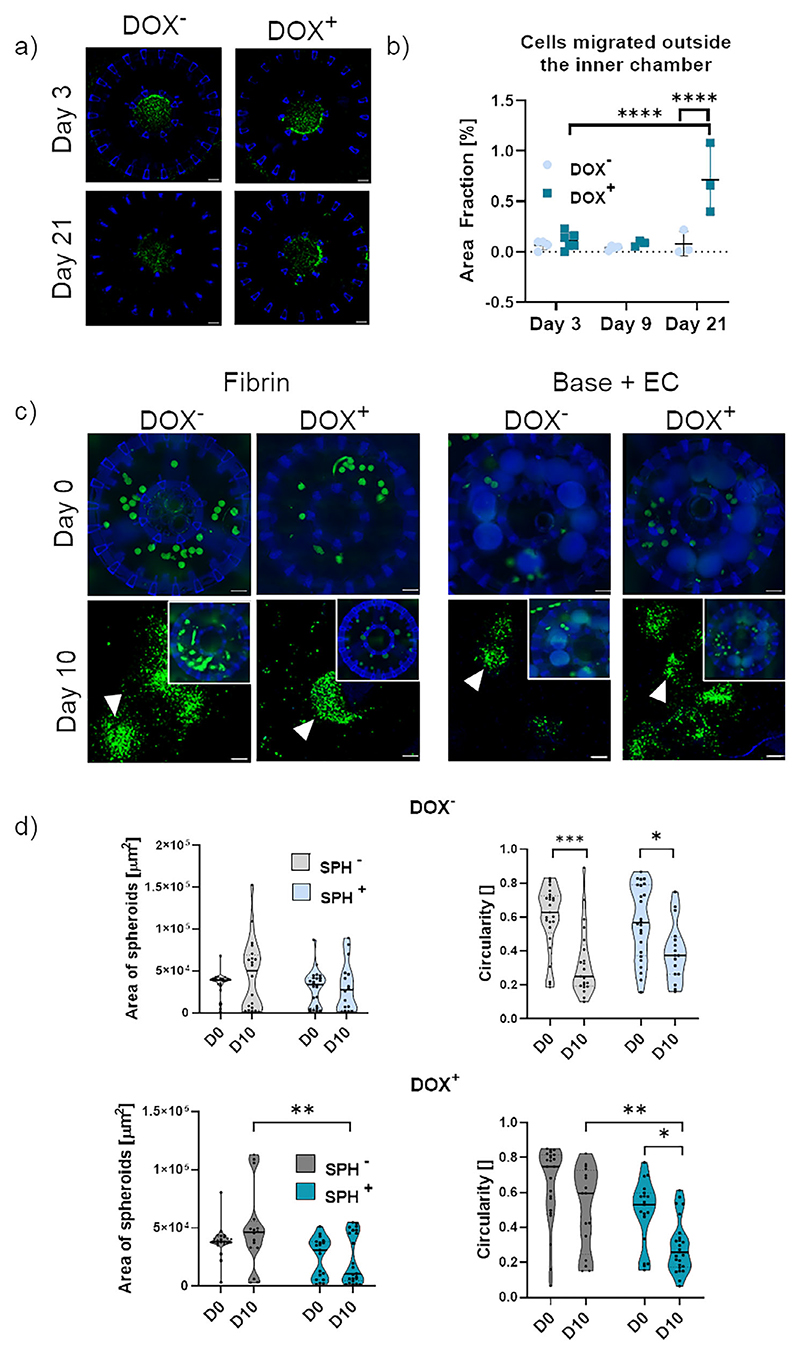
(EwS cell migration. a, b) Single cell migration in Base+EC conditions. *N* = 3 BMSC donors a) Immunofluorescence images of A673/TR/shEF1 ± DOX (green fluorescence) seeded at day 3 of culture inside the inner chamber and imaged at 3, 9, and 21 days. Scale bar = 450 μm. b) Quantification of the area fraction of cell migrated inside the middle chamber. ^****^, *p* < 0.0001, *N* = 6. c,d) EwS cell migration from preformed spheroids in Base+EC versus fibrin gel conditions. c) Immunofluorescence images of A673shEF1 ± DOX cell spheroids (green fluorescence) seeded in the middle chamber of the 3D printed device in the presence of an empty fibrin gel (left) or in the presence of Base+EC conditions (right). *N* = 2 BMSC donors In the upper row, images at day 0; bottom row, images at 10 days, showing magnification of spheroids. White arrowheads indicate spheroids in the magnifications. Scale bar = 450 μm, for the insets scale bar = 200 μm. d) quantification of A673/TR/shEF1 spheroid area and circularity at day10 compared to day 0. P values calculated with two-way ANOVA and Tukey’s multiple comparison test (N spheroids > 15 for each condition). First graph represents spheroid area in the absence of DOX. Second graph represents spheroid circularity in the absence of DOX: ^*^, *p* = 0.0396, ^***^, *p* = 0.0002. Third graph represents spheroid area in the presence of DOX: ^**^, *p* = 0.0032. Fourth graph represent spheroid circularity with DOX: ^**^, *p* = 0.0069, ^*^ = 0.0158.).

Then, we seeded A673shEF1, SK-N_M, and TC71 EwS cell lines in the inner chamber (C) of our device. When only fibrin was present in the B chamber, EwS cells did not migrate, as opposed to SUM159, until day 21, in presence or absence of Dox in the medium (Figure S6, Supporting Information). Seeding A673shEF1in Base+EC model, we observed that Dox induced a significantly higher cell migration at 21 days as compared to no-Dox cells, showing that the presence of an ECO microenvironment fostered the migration of EWSR1::FLI1_low_ but not of EWSR1::FLI1_high_ cells (Figure 6a,b).

Since migration of EwS cells from the inner (C) to the middle chamber (B) was very slow, we also evaluated cell migration from EwS spheroids directly seeded in the middle chamber (B), as shown in the rendering in Figure 2c (bottom right). We compared the behavior of A673shEF1 cell spheroids with and w/o Dox in Base+EC conditions and in fibrin gel (Figure 6c,d). In absence of Dox, we did not observe significant differences in area between days 0 and 10, although circularity significantly decreased, both in fibrin gel and in Base+EC conditions. This indicates movement of cells, leading to a shape modification of the pre-seeded EwS spheroids, with no disaggregation, thus resulting in limited overall migration. The addition of Dox in the Base+EC condition caused a significant decrease in spheroid circularity and area at 10 days as compared to day 0 and to fibrin gel. Thus, the addition of Dox led to spheroid disaggregation (inset in Figure 6c) in Base+EC condition, suggesting a more migratory phenotype of EWSR1::FLI1_low_ cells. Overall, our data demonstrated that a microenvironment reproducing initial ECO hallmarks was able to promote the invasive phenotype of EWSR1::FLI1_low_ cells, fostering their migration both as single cells as well as from preformed spheroids toward the microenvironment itself.

## 3 Discussion

In our work we combined different 3D culture techniques, such as cell spheroids, 3D printed supports and self-assembled vascularization, all of which have shown promising results for the reproduction of complex 3D tissues, ^[ 35–37]^ aiming at achieving an in vitro model of ECO. Our approach allows overcoming some of the limits of present microphysiological systems, combining compartmentalization typical of microfluidic devices with real 3D structure and higher scale of spheroids and tissue engineered constructs. ECO multi-step process starts with the condensation of mesenchymal progenitors, which we recapitulated through the formation of MSCs spheroids or micro-masses (SPHs). After condensation, ECO progresses with a chondrogenic phase, during which the transcription factors SRY-box containing gene 9 (*SOX9*), *SOX5* and *SOX6* are upregulated to achieve MSC differentiation toward chondrogenic phenotype^[24]^ and the subsequent deposition of an Extra-cellular matrix (ECM) rich in Glycosaminoglycan (GAG) and collagen type 2 (Col2).^[38]^ In our model, and in particular within chondrogenically differentiated SPHs, we were able to recapitulate the activation of transcription factors and deposition of cartilage-like ECM, providing a template for the following hypertrophic step. In this second ECO phase, hypertrophic chondrocytes express Matrix Metalloproteinase 13 (MMP13), Collagen type 10 (Col10), and vascular endothelial growth factor (VEGF).^[22]^ In our hypertrophic SPHs, 14 days of differentiation triggered an almost significantly higher the expression of Col10 at RNA, with an increasing trend for all the patients analyzed. Although an early upregulation of Col10 expression in BMSCs cultured for 14 days has been reported,^[39]^ culture periods as long as 21 days have been used for fully hypertrophic differentiation in vitro of BMSCs pellets.^[32]^ In our system, prolonging SPHs culture for 7 days within a 3D microenvironment induced further progression of the ECO process, only when HUVECs were included in the microenvironment surrounding the SPHs. Indeed, the presence of CaPN did not increase the expression of COL10 and MMP13, and did not decrease the expression of SOX9 and COL2, suggesting that calcium phosphate nanoparticles did not promote ECO progression.

This is similar to what was shown in vivo in a fracture healing model based on hydroxyapatite scaffolds, in which hydroxyapatite presence inhibited ECO process progression.^[33]^ Interestingly, also upregulation of RUNX-2, a necessary factor for ECO progression,^[40]^ was achieved only in presence of HUVECs, further supporting the key role of vascular invasion in the hypertrophic zone for longitudinal bone growth.^[41]^ Vascularization is a fundamental step in ECO process, since vascular invasion, promoted by the activity of transcription factors like *RUNX2* and Indian Hedgehog (*IHH*) in hypertrophic chondrocytes, can then stimulate the migration of bone cells, which use the cartilage matrix as template for bone deposition.^[34]^ Endothelial cells invade the cartilage template attracted by angiogenic factors (i.e., VEGF) released by hypertrophic chondrocytes^[3]^ and, to achieve this, current ECO engineered constructs, after an in vitro differentiation phase, rely on in vivo implantation.^[6,7,42]^ However, with this approach, template vascularization is performed by mouse host cells, obtaining models that are not completely humanderived. In addition, within an in vivo setting it would have been impossible to decouple the effects of vascularization and mineralization on EwS progression. To overcome these limitations, we chose an in vitro pre-vascularization strategy, leveraging a 3D printed support structure, that allowed the seeding of an endothelial monolayer and the subsequent sprouting of new vessels into the hypertrophic cartilage template, recapitulating the formation of a vascular network around spheroids, as already demonstrated with pre-vascularized scaffolds based on assembled spheroids for bone augmentation.^[43]^ In our system, VEGF production by SPH embedded in the gel contributed to HUVECs migration in the gel. The decreased content of VEGF in the presence of HUVECs can be attributed to the expression of VEGF decoy receptor FLT-1 by the HUVECs themselves. In the literature it has been already shown that in co-cultures of bone derived cells and HUVECs, the presence of VEGF either supplemented in the culture medium^[44]^ or produced by co-cultured cells,^[45]^ increases the expression of both FLT-1 and KDR in HUVECs, as we also observed in our conditions.

After achieving an in vitro model, reproducing the main hallmarks of the ECO process, such as the expression of COL10, MMP-13, RUNX2, the deposition of collagen X in the matrix and the ingrowth of vascular structures, in a different extent depending on the model constituents, we wanted to investigate the behavior of EwS cells in this microenvironment. In particular, we aimed to compare tumor cell growth in different stages of the ECO process, trying to elucidate the influence of different developmental phases on EwS progression. The seeding of single tumor cells in our devices, at different degrees of maturation, showed that a more hypertrophic vascularized environment induced an increase in EwS clusters dimensions, indicative of a higher cell proliferation. The contribution of vasculogenesis to the growth of EwS tumors was already described some years ago, using a transgenic mouse model lacking vascular progenitor cells and injected with human EwS cells.^[46]^ In this study, the presence of normal vessel structures at the tumor site was required for EwS tumor growth, although injection of the tumor cells was performed subcutaneously, thus lacking the biophysical stimuli deriving from a bone environment. Cells present in bone microenvironment produce growth factors such as VEGF, which has been described as a key factor for the recruitment of new endothelial cells from the bone marrow fostering tumor growth.^[47]^ In our device, SPHs and cells included in the model produced VEGF, which stimulated vessel formation and invasion of the hypertrophic microenvironment. However, the lower content of VEGF in Base+EC constructs as compared to other conditions suggests that other endothelial cells related factors can be involved in the increased tumor growth. Beyond secreted factors, it has been shown that tumor progression depends on the composition and structure of the extracellular matrix (ECM).^[48]^ In EwS patients, highly aggressive subtypes with Wnt-*β* catenin activation overexpress several ECM genes, as compared to more quiescent tumors, and in particular a group of genes called Angiomatrix^[49]^ is greatly upregulated. The increased expression of these genes is associated to the angiogenic switch, a process essential for successful expansion of the tumor but also for metastatic growth and reactivation of dormant phenotypes.^[50]^ Interestingly, among the genes more expressed in the Angiomatrix, there are *Col10* and *MMP13*,^[51]^ which are expressed at higher levels in Base+EC as compared to other conditions. higher content of these Angiomatrix proteins in the tumor microenvironment can be suggestive of a potential angiogenic switch in tumor cells, indicating a more aggressive and proliferative tumor stage, as reported.^[49]^ Surprisingly, the addition of CaPN in the models including SPHs led to a decreased growth of EwS clusters, whilst in absence of SPHs this effect was not observed. This suggests that the presence of CaPN per se did not inhibit EwS cell proliferation and highlights how increased complexity within a microphysiological system allows evidencing otherwise neglected microenvironmental effects. It can be speculated that CaPN affected further progression of late hypertrophy as happens during ECO, hence decreasing the production of factors such as Col10 and RUNX-2. Indeed, it has been reported that in a rat model of bone healing, leveraging nanohydroxyapatite loaded scaffolds, that the presence of nanoparticles inhibited the formation of new bone through ECO as compared to scaffolds without nanoparticles, leading to the direct ossification of the scaffold periphery.^[33]^

Finally, we preliminary looked at the influence of the hypertrophic vascularized environment on EwS cell migration, starting to explore the behavior of differently migrating EwS cells. The single cell migration of EWSR1::FLI1high cells into Base + EC model did not occur even at 21 days, when instead migration of EWSR1::FLI1low cells started, confirming that decreased expression of the fusion gene led to a more aggressive phenotype also within an ECO microenvironment, comparable to the behavior observed in 2D and in 3D collagen gels.^[13]^ Furthermore, the migration from pre-formed spheroids was significantly increased in the ECO environment as compared to fibrin gel, whereby the disaggregation of spheroids at 10 days was negligible even in the presence of Dox. This increased migratory phenotype in a bone-like environment could be due to the upregulation of ECM genes, which promoted a metastatic phenotype in EwS cells, by creating EWSR1::FLI1low-like cell states.^[52]^ In this context, it has been shown that Col10 expression is elevated in tissues from sarcoma samples^[53]^ and other tumor tissues of different origin (e.g., prostate,^[53]^ lung^[54]^ and breast^[55]^), and has been correlated with a worst overall survival of patients, indicating a more aggressive phenotype. Interestingly, also MMP13 has been reported as one of the most expressed genes in human osteosarcomas, promoting tumor proliferation^[56]^ and metastasis.^[57]^ Considering the variable expression of these factors depending on the model composition, it could be inferred that a proper microenvironment, mimicking as closely as possible the developmental pathways activated in vivo, is needed to activate proliferative and invasive behavior in EwS cells.

## 4 Conclusion

In conclusion, our data suggest that factors present in the vascularized hypertrophic cartilage microenvironment, including ECM proteins, can be associated with increased tumor cell proliferation and migration. Further investigations are needed to elucidate if increased proliferation and migration are due to the activation of pathways promoting EwS cell aggressive phenotypes.

## 5 Experimental Section

### Cell Culture

Bone marrow-derived mesenchymal stem/stromal cells (BMSCs) were donated by the Department of Biomedicine (University of Basel, Basel, CH) or isolated from exostosis harvested at the orthopedic unit of the Istituto Pediatrico della Svizzera Italiana (Ente Ospedaliero Cantonale, Bellinzona, CH). All biopsies used to isolate the BMSCs were taken from anonymous young healthy donors (2 males, age 17,5 ± 0,5, 2 females, age 21 ± 7). Anonymous donors, or their family on their behalf, gave their consent for the use of obtained biological material. Cells were isolated from bone marrow or spongiosa rinsed in PBS and centrifugated (510 g for 10 min). Cells were expanded in Alpha-MEM with 10% inactivated Fetal Bovine Serum (FBS, Hyclone), 1% Hydroxyethyl Piperazine Ethane Sulfonic acid (HEPES, GIBCO), 1% Pen Strep (PS, GIBCO) and with the addition of 5 ng/ml human Basic Fibroblast Growth Factor (FGF2, Peprotech). Cells isolated from four patients were expanded and used for the experiments at passage 4: 2 males and 2 females with median age 17,5 (range 14–28). Green fluorescent protein (GFP)-transfected Human Umbilical Vein Endothelial Cells (HUVECs) were purchased from Angio-Proteomie, expanded in Endothelial Growth medium (EGM-2, Lonza) with addition of 3% of FBS up to passage 7. Human Ewing sarcoma (EwS) cell lines (TC-32 and A673shEF1) were provided by the Division of Translational Pediatric Sarcoma Research (German Cancer Research Center (DKFZ), Hopp Children’s Cancer Center (KiTZ), Heidelberg, DE). Cells were cultured in Roswell Park Memorial Institute medium (RPMI, Life Technologies) supplemented with 10% FBS and 1% PS. The EWSR1-FLI1 knockdown was achieved by adding 1 μg/ml of doxycycline (DOX) every 48 h to culture medium.

### Hypertrophic Spheroids Differentiation

To form BMSC hypertrophic SPH, 96 multi-well plates were coated with a 2% solution of poly-2-hydroxyethyl methacrylate (poly-HEMA) to prevent cell attachment and promote cell aggregation. Poly-HEMA was prepared by putting bain-marie a solution of absolute ethanol with 5% distilled water and 200 mg of poly-HEMA (Sigma) on a magnetic-stirrer at 70 °C. 50 μl of solution were poured in each well and the 96 well plates were left overnight under the hood to allow ethanol evaporation. BMSCs were then seeded in the coated well plates at a concentration of 0.025 × 10^6^ cell well^−1^ and cultured for 14 days in chondrogenic medium composed by Serum Free Medium (1% HEPES, 1% sodium pyruvate 100X (GIBCO), 1% PS, 1% L-Glutamine (GIBCO), 1% Insulin, Transferrin, Selenium +1 (ITS+1, Sigma) and 1.25 mg mL^−1^ Human Serum Albumin (HSA, Sigma), enriched with 0.1 mm L-Ascorbic Acid 2-Phospahte (Sigma), 0.1 μm Dexamethasone (Sigma), 10 ng mL^−1^ Transforming Growth Factor-*β*1 (TGF-*β*1, Peprotech). After 14 days (Day 14 in Figure 2a), the medium was switched to hypertrophic medium to reach hypertrophic differentiation of the cell aggregates. Hypertrophic medium (HyM) is composed by Serum Free Medium enriched with 10 mm *β*-glycerophosphate (Sigma), 0.05 μm L-Thyroxine (Sigma), 0.1 mm Ascorbic Acid 2-Phospate, 0.01 μm Dexamethasone. Cellular aggregates were cultured for 14 days in HYP medium (Day 28 in Figure 1a). Culture medium was changed every three days. For preliminary evaluation of BMSC differentiation capability, BMSCs in aggregates were differentiated toward the chondrogenic or osteogenic phenotype (osteogenic medium: DMEM, 10% FBS, 2 mm L-glutamine, 1 mm sodium pyruvate, 10 mm HEPES, 1% PS supplemented with 0.01 μm Dexamethasone, 10 mm *β*-glycerophospate, 10 nm cholecalciferol, 150 μm L-ascorbicacid-2-phosphate) for 10 days. Controls were prepared by culturing aggregates with respective media without growth factors.

### Calcium Phosphate Nanoparticle (CaPN) Synthetization and Characterization

Calcium Phosphate Nanoparticles (CaPN) were synthetized following a previously developed protocol.^[30]^ A suspension of CaP nanoparticles was prepared using a wet-chemical precipitation method, according to the following reaction between calcium hydroxide (Ca(OH)2) and orthophosphoric acid (H3PO4): 5*Ca*(*OH*)2 + 3*H*3*PO*4*Ca*5(*PO*4)3(*OH*) + 9*H*2*O*. Briefly, 25 mL of H3PO4 solution (3.56 m, Acros) was added dropwise into 25 mL of Ca(OH)2 (5.92 M, Acros), under continuous stirring at 60 °C for 15–18 h. After aging, CaPN were rinsed three times through centrifugation (2800 × g, 2 min) and were resuspended with deionized water at a concentration of 100 mg mL^−1^. The suspension was sterilized by autoclaving and the pH was adjusted to 7.4. After drying, CaPN were characterized by scanning electron microscopy and with energy dispersive X-ray spectroscopy. Nanoparticles were dried and placed on an aluminum foil for acquisition.

### 3D Printed Device Fabrication

Aiming at recapitulating the complex ECO environment, whereby hypertrophic cartilage template is invaded by vascular cells and then mineralized, a custom compartmentalized 3D device was designed, allowing to reproduce the migration of vascular cells into a matrix embedding hypertrophic SPH (contained in chamber B). Furthermore, since leverage would the system to study the migration of EwS cells, the system was provided with a further chamber (C) for cancer cell seeding. The 3D printed device was designed using a CAD software and its final configuration and dimensions are reported in Figure 2b and Figure S7 (Supporting Information). The walls separating the chambers have square pores (0,45 mm each side) to allow cell migration between the compartments and the external chamber acting as a medium reservoir (A) contains up to 300μL. Devices were printed by digital light processing (DLP) printing with a commercial biocompatible-certified and transparent resin (GR-10 by pro3Dure). After printing, the excess resin was washed from the devices with two isopropanol rinsing in an ultrasound bath and postcured by an ultraviolet (UV) light flashing to complete object polymerization. The devices were glued with a silicone-glue to 18mm-diameter glass slides. The devices were sterilized in isopropanol for 20 min, rinsed twice with demi-water, and left for 1 h under UV light. (Figure 2c).

### Optimization of Co-Culture Conditions in Device—Definition of Medium Composition

Considering the different cell types involved in the model, the culture medium had to be adapted to guarantee cell survival and continuous differentiation of hypertrophic spheroids. Medium composition was chosen among different combination: EGM2, as standard condition, hypertrophic medium (HypM) and a combination of EGM and HypM (1:1). Devices were seeded with 40 μL of 10 mg mL^−1^ fibrin gel with different combinations of cells: a monoculture of HUVECs at a concentration of 3 × 10^6^ cell mL^−1^, and a co-culture of HUVECs and BMSCs (3 × 10^6^ cell mL^−1^ HUVECs + 1.5 × 10^6^ cell mL^−1^ BMSCs). Devices were covered with 400 μl of medium, that was changed every other day. Metabolic activity of cells was measured at day 3, 5, and 7 with Alamar blue. Briefly, a 10% v/v Alamar blue solution was added to samples in triplicate and the absorbance signal (570 nm/600 nm) was detected after 2 h of incubation at 37 °C (Tecan Infinite M Plex). In addition, at day 7 a LIVE/DEAD assay was performed and pictures were taken with a fluorescence microscope (Nikon Eclipse TI).

### Definition of CaPN Density

To determine potential cytotoxicity of CaPN enriched fibrin gel, four different concentrations of CaPN were tested (0, 2.5, 5, 7.5 mg mL^−1^). In more details, 40 μL of fibrin gel at a concentration of 10 mg mL^−1^, enriched with CaPN and embedding cells was seeded a 96 well plate and covered with 200 μL of medium. Three different cell combinations were tested: monoculture of 3×10^6^ cell mL^−1^ HUVECs, monoculture of 1.5×10^6^ cell mL^−1^ BMSCs and co-culture of 3×10^6^ cell mL^−1^ HUVECs and 1.5×10^6^ cell mL^−1^ BMSCs. The medium was refreshed every other day. LIVE/DEAD assay was performed at days 3, 5, and 7 and fluorescent images were taken and area fraction of green and red signal has been quantified (see below).

### Seeding of Endochondral Ossification (ECO) Constructs

After optimizing the culture conditions to reproduce the different ECO stages, the model elements in various ways were combined: SPHs at the hypertrophic stage, CaPN nanoparticles, and the HUVEC monolayer. More in detail, for all conditions, at day 0 ≈20 SPHs (this number enables a homogeneous distribution when filling the middle chamber) and 1.5 × 10^6^ cells mL^−1^ undifferentiated BMSCs (to support the formation of the vascular network, as already shown in the previous work^[30]^) and HUVECs (6 m mL^−1^) for conditions Base+EC and Base+EC+CaPN were suspended in EGM2 medium with 4 U mL^−1^ thrombin (Baxter), mixed with a solution of 20 mg mL^−1^ of fibrinogen and injected in the middle chamber of the device (Figure 2d). Final fibrin gel concentration was set at 10 mg mL^−1^ to avoid gel shrinking from the top of the device, based on preliminary tests performed (results not shown). This represented the Base condition, mimicking the first ECO stage (chondrocyte hypertrophy). In a different condition CaPN (Base + CaPN) was added in the optimized concentration, to mimic a more advanced ECO mineralization stage. All the devices were put in a humidity chamber and left in incubation at 37 °C for 20 min, moved in a 12 multiwell plate and 400 μL of EGM2+HypM medium were added. To mimic the following vascular invasion process, a monolayer of endothelial cells was formed on the external surface of the middle chamber at day 1, seeding HUVECs with a concentration of 2 × 10^6^ cell mL^−1^ in EGM2. On each side of the middle chamber 50 μL of cell suspension was deposited and left in incubation for 30 min. Then the plates were tilted and the second side was seeded. After 1 h, excess cells were removed and culture medium was added. HUVECs were left sprouting in the matrix with SPHs in fibrin (Base + EC), reproducing the initial invasion of the cartilage template, or in fibrin with nanoparticles (Base + CaPN + EC) resulting in a more mature bone stage. All the cultures were then maintained for 7 days and the medium was replaced every other day.

### Histological and Immunofluorescent Staining—BMSC Immunofluorescent Staining

The BMSCs isolated from the biopsies were cultured in 6-well plates. Undifferentiated BMSCs or BMSCs differentiated for two weeks in osteogenic medium (see paragraph Hypertrophic spheroids differentiation) were compared. After confluence, cells were fixed for 10 min with 2% paraformaldehyde (PFA) and rinsed twice with PBS. BMSCs were permeabilized with 0.1% Triton-X 100 in PBS for 30 min at 37 °C under stirring. 4% BSA solution in PBS was added to block unspecific binding for 1 h at 37 °C. After blocking, samples were incubated with primary antibodies for 1 h under stirring at 37 °C. After two rinsing with PBS, secondary antibodies with 3 drops mL^−1^ NUCBLUE-DAPI (ThermoFisher) were added to the samples for 1 h under stirring at 37 °C. Images were taken using a confocal microscope (Nikon C2). Cells were stained with 10 μg mL^−1^ anti-CD73 to assess their mesenchymal origin (ThermoFisher). Primary antibodies used to assess osteodifferentiation were 10 μg mL^−1^ osteocalcin monoclonal antibody (ThermoFisher), 5 μg mL^−1^ osteopontin monoclonal rabbit antibody (ThermoFisher) and 1 μg mL^−1^ Collagen I monoclonal mouse antibody (ThermoFisher). The secondary antibody for these primaries was AlexaFluor 488 antirabbit or antimouse (ThermoFisher).

### Histology of SPHs

SPHs at day 14 and 28 of differentiation were fixed in 4% paraformaldehyde (PFA) solution overnight at +4 °C, then samples were rinsed twice with PBS and were stored at +4 °C for further experiments. For Optimal Cutting Temperature compound (OCT) embedding, samples were put overnight in a 4% Sucrose (Sigma) solution in PBS. Then, samples were washed with PBS, embedded in OCT (CellPath) and left for 20–30 min at room temperature to allow the penetration of OCT in the sample. 10 μm thick sample slides were obtained by cryostat-cutting (ThermoFisher). Samples were stained for Masson’s Trichrome, Alizarin Red and Safranin O staining. The images were taken with a color camera widefield microscope (Nikon DS-Fi2).

### Staining of the Constructs

Devices were fixed after 7 days of culture in 4% PFA for 3 h at room temperature and stored at 4 °C. Samples were permeabilized and blocked with 0.1% Triton-X 100 in 4% BSA for 1 day at 4 °C under stirring. Primary and secondary antibodies were incubated for 1 day at 4 °C under stirring.

Primary antibodies used were Collagen II monoclonal mouse antibody (MA5-12789, ThermoFisher Scientific), Collagen X monoclonal mouse antibody (ab49945, AbCAM), with a final dilution of 10 μg mL^−1^ in 4% BSA. The secondary antibody was 2 μg mL^−1^ AlexaFluor 568 goat anti-mouse (A11011, ThermoFisher Scientific) with with 3 drops mL^−1^ NUCBLUE-DAPI (ThermoFisher). Before acquisition at the confocal microscope samples were put in RapidClear (SunjiLab) tissue clearing solution for 1 h.

### GAG Quantification

SPHs at day 1,7, 14, 21, 28 were digested in 1 mg mL^−1^ K proteinase solution at 56 °C overnight and stored at −20 °C. DNA quantification was done spectrofluorometrically by CyQUANT assay (Invitrogen) on triplicate with calf thymus DNA as a standard and according to the manufacturer’s instruction. The fluorescence signal (480 nm/520 nm) was detected with a plate reader (Tecan Infinite MPlex). The glycosaminoglycans (GAG) quantification was measured by 1,9-dimethyl-methylene blue (DMMB) assay in triplicate. Plate was read with an absorbance of 535 nm. The GAG content was normalized to the DNA content in all samples.

### Gene Expression Analysis

SPHs at day 1,7, 14, 21, 28 of differentiation, and 7 days-co-culture samples were analyzed for gene expression analysis using real-time PCR. Before the RNA extraction, samples were homogenized using a sonifier (BRANSON). Subsequently, the RNA was extracted using the ReliaPrepTM RNA Cell Miniprep System (Promega) following manufacturer’s instructions. RNA concentration and quality were measured using NanoDrop ND100 UV–vis spectrophotometer (ThermoFisher). cDNA was prepared obtained using Quanti-Tect Reverse Transcription Kit (Quiagen) according to the manufacturer’s instructions. Briefly, the reverse transcription was carried out at 42 °C for 10 min, followed by 15 min after the addition of the template RNA. The reaction was stopped by heating for 3 min at 95 °C. Subsequently, qRealtime (RT)-qPCR was performed in a 10 μL reaction mix containing Taqman Universal PCR master mix (Thermo Fisher, Zürich, Switzerland), the selected primers/probes, DEPC-treated H2O and the cDNA template. The relative gene expression of *COL2A1* (Hs01060344_g1) and *SOX9* (Hs00165814_m1) as chondrogenic genes (week 2) and *RUNX2* (Hs00231692), *COL10A1* (Hs00166657_m1) and *MMP13* (Hs00233992_m1) as hypertrophic genes (week 4), of *COL1* () and *BGLAP* () as osteogenic genes and of *FLT-1* (Hs01052961_m1) were calculated as 2(-ΔΔCt)by using ribosomal protein large, P0 (*RPLP0*, Hs00420895_gH) as endogenous control. Fold change calculation was specified in captions of related graphs. The reaction was set up as follows: 50 °C for2 min, 95°C for 10 min and 40 cycles of 95 °C for 15 s followed by an annealing/extension step at 60 °C for 1 min. All the qPCR runs were performed using a CFX Real-Time PCR System (Bio-Rad). Technical replicates were used for each target gene and the different donors (biological replicates).

### Vascular Endothelial Growth Factor (VEGF) Release in Devices

To measure the release of human Vascular Endothelial Growth Factor (VEGF) during co-culture, culture media were collected from devices in different conditions (Base, Base + CaPN, Base + EC, and Base + EC + CaPN) at days 1, 4, and 7 and immediately frozen at −80 °C. Conditioned medium from SPHs after 28 days of differentiation, EGM2 and EGM2+HypM were used as controls. A solid phase sandwich ELISA was performed by using Human VEGF DuoSet ELISA kit (R&DSystems) following manufacturer instructions. This assay has a sensitivity of 31.2–2000 pg^−1^. Six samples were tested for each condition and all samples were tested in duplicate.

### Proliferation of EwS Cells in the Devices

To perform proliferation assays on EwS cells, the TC-32 cell line was used, which was transduced with a tdTomato red fluorescent vector. The cells were seeded inside the middle chamber at day 0, mixing them with SPHs and BMSCs in fibrin solution as described above at a concentration of 0.075×10^6^ cell mL^−1^. To study the effect of different stages of ECO microenvironment on TC-32 behavior, TC-32 seeded on Base was compared, Base+CaPn, Base+EC, and Base+EC+CaPn, obtained as described above. To check for the effects of SPHs steric hindrance, the results were compared with the same environment (i.e., only BMSCs, addition of CaPN and /or HUVEC) but w/o SPHs. All the samples were kept in culture for 7 days and media was changed every two days. At the end of culture samples were fixed and imaged under a confocal microscope (Nikon C2), after counterstaining with DAPI, as described before. Cell growth in the different ECO environment has been measured through quantification of the red-fluorescent area in the different images (*n* = 6 samples for each condition) as described below.

### Migration of EwS Cells in the Devices

The migratory potential of different EwS cell lines was evaluated. First, a standard 2D scratch assay was performed. Briefly, Dox-inducible A673/TR/shEF1 cells were seeded in 12-well plates and allowed to reach confluence. Then, a scratch was made with a 10 μL pipette tip. Wells were washed with PBS to remove detached cells. To investigate the effect of EWSR1::FLI1 expression on cell migration properties, medium was enriched with 1 μg mL of Dox to silence its expression. Cells were kept in culture for 24 h or until the wound was closed. Pictures were taken at 0, 8, and 24 h. Afterward, a single-cell 3D migration assay in the device was performed. Briefly, all the three cell lines were seeded in the C chamber two days after seeding of the B chamber with the Base+EC conditions as described above or with only fibrin at the same concentration (10 mg mL^−1^ final) for control. First, the seeding density comparing five different values were optimized: 0.125 × 10^6^, 0.312 × 10^6^, 0.625 × 10^6^, 1.25 × 10^6^, and 1.5 × 10^6^ cells mL^−1^. Cells were stained with Vybrant Dil (Thermofisher) to allow cell visualization during culture and were pre-treated with 1 μg mL^−1^ of Dox 48 h before the seeding in the device for silencing EWSR1-FLI1. As a positive control, devices were seeded with breast cancer cell line SUM-159. To monitor cell migration from the C chamber to the B chamber, pictures were taken (*n* = 3 samples for each condition) after the seeding of the C chamber and on several time points up to day 22. Finally, a further migration experiment was performed, seeding spheroids of A673/TR/shEF1 directly in the B chamber of the devices. After A673 spheroid generation, fibrin was mixed with at 10 mg mL^−1^ or with the components of the Base+ECO condition and injected them in the B chamber of the device. In incubation for up to 10 days was left them and imaged the samples at days 0 and 10. Area and circularity of A673 spheroids were quantified from confocal images as described below.

### Image Quantification

For quantification of the fluorescent area, used for live&dead, proliferation and migration experiments, images were analyzed using Fiji software. All analyzed images were pre-processed to subtract the background (rolling ball radius = 50 pixels) and to adjust the contrast and brightness levels. Then, images were converted to a binary format by applying a threshold. The segmented image, showing in white the green signal (i.e., calcein in live&dead experiments or Vybrant DiO in cell migration experiments) or the red signal (td-Tomato in cell proliferation or Vybrant DiL in cell migration experiments), was created and different parameters (area fraction, area, diameter, circularity of single particles) were measured with the Analyze Particles plugin. Area fraction was defined as the ratio of the white (i.e., signal) over the black area (i.e., background).

### Statistical Analysis

Graph Pad Prism 8.0.2 software was used to perform statistical analysis on raw data. If not specified otherwise in the figure legends, unpaired Student’s t-test or one-way ANOVA were performed to determine significant differences between conditions. Differences were considered significant for *p* < 0.05 (*), *p* < 0.01 (**), *p* < 0.001 (***), and *p* < 0.0001 (****). Results are presented as mean ± standard deviation. Details about P values and the number of replicates are reported in each figure legend.

## Supplementary Material

Supporting information

## Data Availability

The data that support the findings of this study are openly available in [Open Science Framework] at https://doi.org/[DOI10.17605/OSF.IO/NQ6GA], reference number [4].
