# Supplementary material for: A Vascularized Microphysiological System Reproducing Endochondral Ossification in Vitro to Study Ewing Sarcoma Proliferation and Migration
Source: Adv Funct Mater. Author manuscript; Available in PMC 2026 May 29. (PMC7619114; doi:10.1002/adfm.202418470)
Supplement: Supporting information [file EMS213802-supplement-Supporting_information.docx]

Supporting Information

A Vascularized Microphysiological System Reproducing Endochondral Ossification in Vitro to Study Ewing Sarcoma Proliferation and Migration

*Maria Vittoria Colombo, Chiara Arrigoni*, Tobias Faehling, Antonietta Verrillo, Viviana Secci, Thomas G P Grünewald, Giuseppe Talò, Alexandra Kummer, Jorge Gonzalez, Vincenzo De Rosa, Christian Candrian, Andrea Barbero, Florencia Cidre-Aranaz and Matteo Moretti*


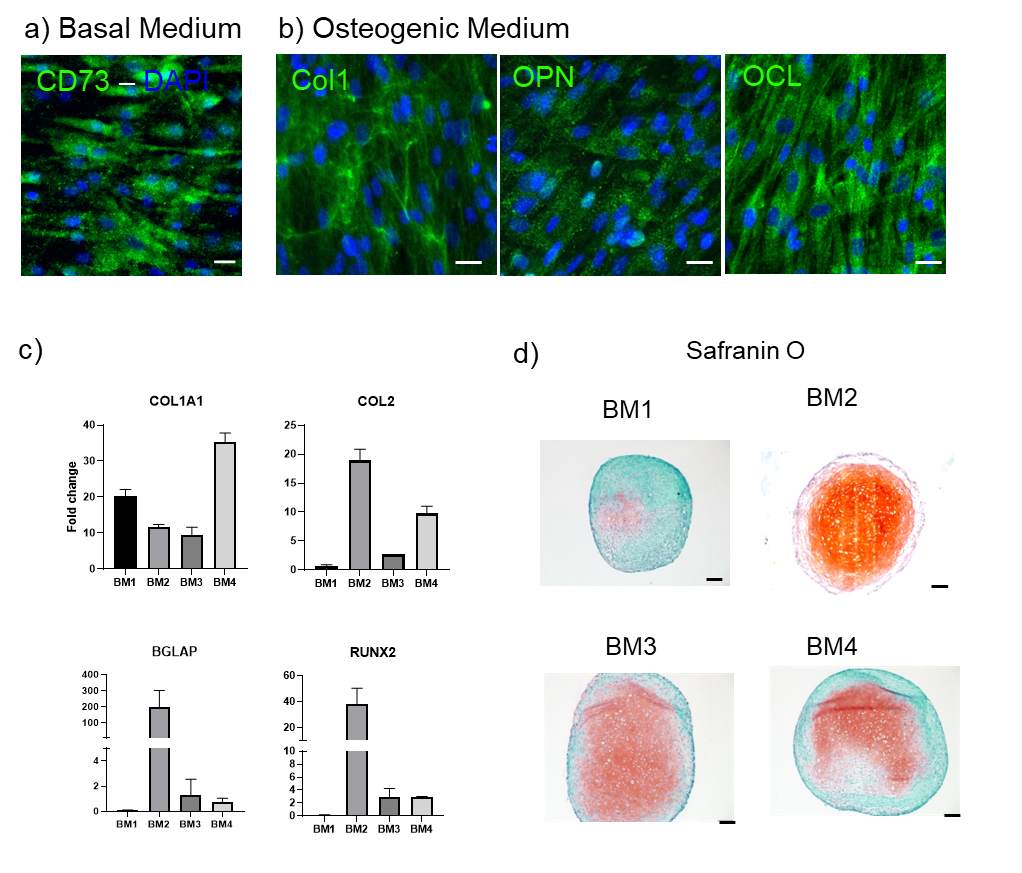


**Figure S1. (**BMSC characterization. a) Representative image showing the expression of CD73 (green) after isolation and culture in growth medium. b) BMSCs in 2D after 10 days of differentiation in osteogenic medium express collagen 1 (Col1, green), osteopontin (OPN, green) and osteocalcin (OCL, green). Scale bar=10 μm. c) Gene expression analysis of chondrogenic and osteogenic genes in pediatric BMSCs. Chondrogenic genes (COL2A1, COL1A1) were evaluated in spheroids whilst osteogenic (BGLAP, RUNX2) in 2D cultures. ΔCt is normalized over GAPDH as average of n=3 samples. The fold change was calculated as differentiated spheroids and 2D cells over undifferentiated cells. d) Representative images showing the results of chondrogenic differentiation of pediatric BMSCs in 3D spheroids: Safranin O staining (GAG matrix, red) with a Fast green counter staining. Scale bar=50 μm.


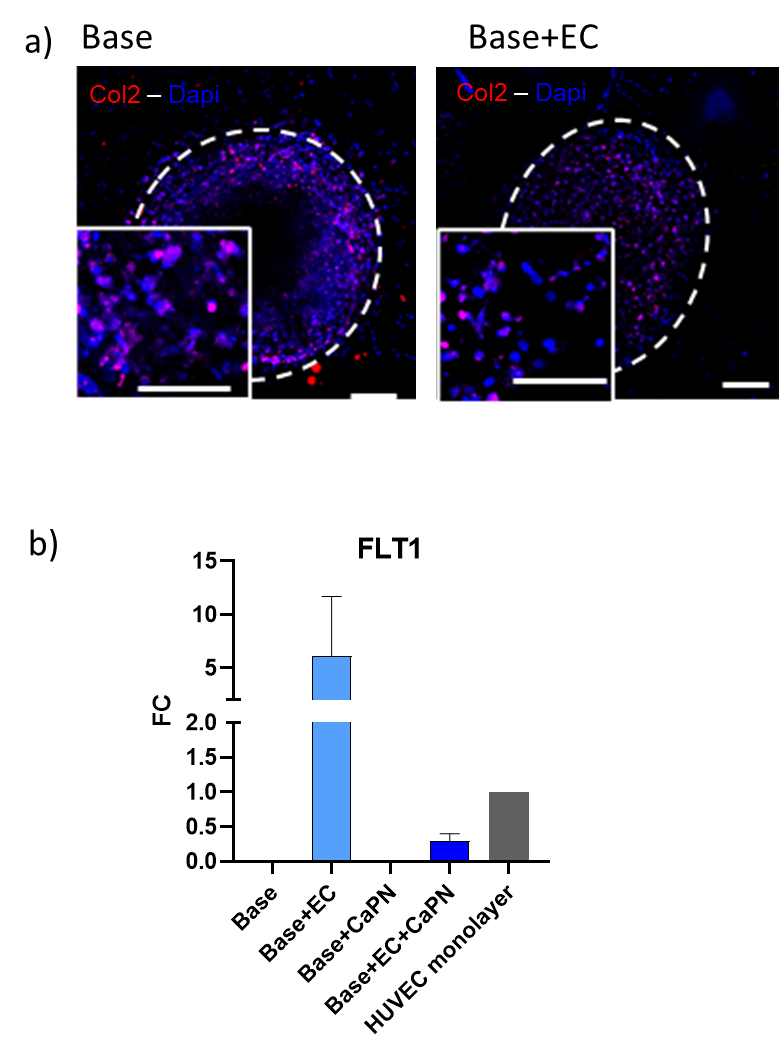


**Figure S2.** (a) expression of Collagen 2 in Base and Base+EC conditions, showing nuclear expression of the markers in both conditions. Scale bars 50 um. b) Expression of VEGF decoy receptor FLT-1 in devices in different conditions and in HUVECs cultured in 2D monolayer for comparison.


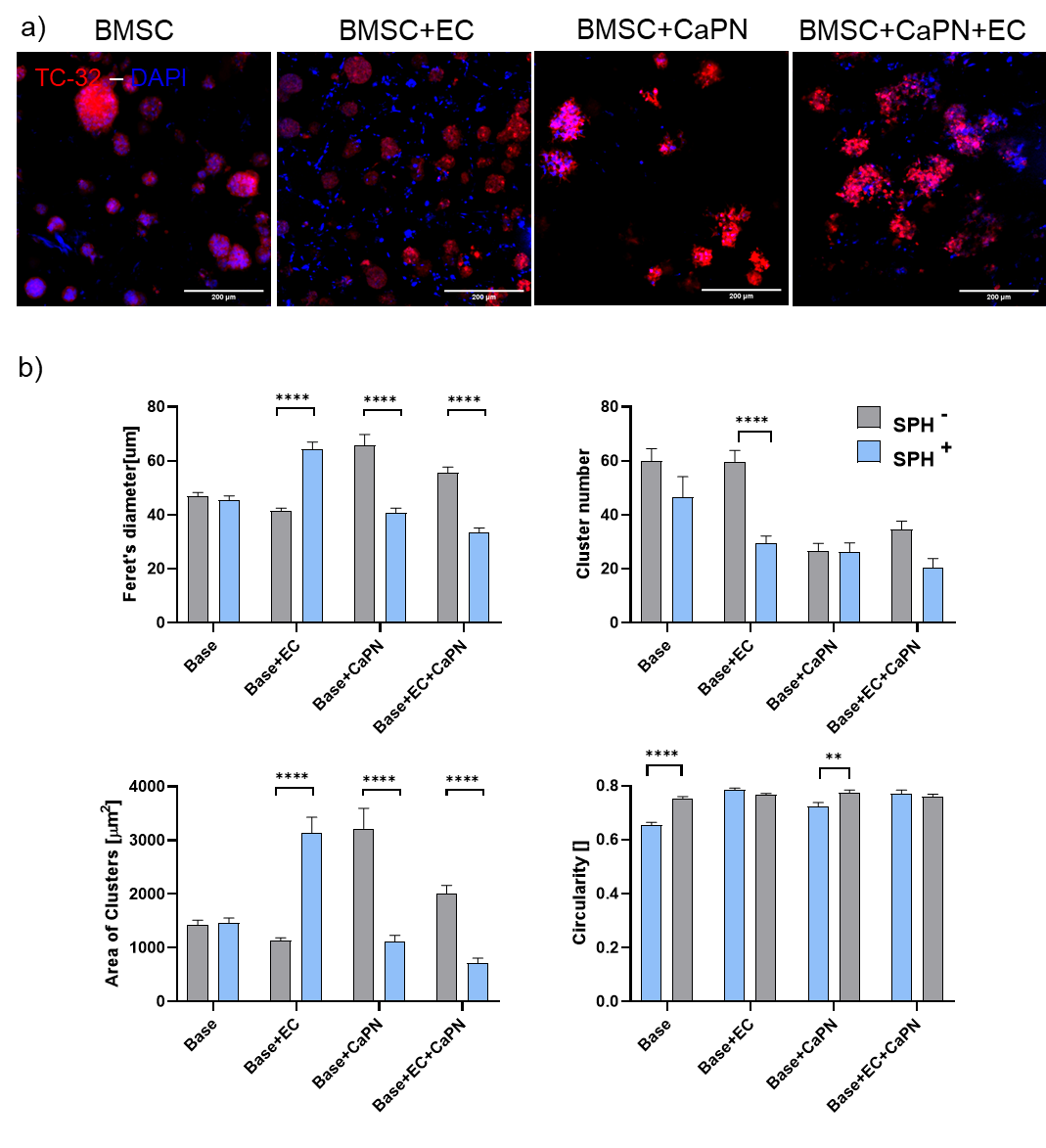


**Figure S3.** (EwS cell proliferation in a microenvironment without SPHs. A) Immunofluorescence images of TC-32 cluster (red fluorescence, nuclei in blue) formation after 7 days of co-culture in the middle chamber with fibrin gel, BMSCs and w or w/o CaPN and H. Scale bar=200 μm. b) Quantification of TC-32 cultured in devices in presence (SPHs) or absence (No_SPHs) of SPHs. P values calculated with two-way ANOVA and Sidak multiple comparison test (n=6 samples per condition). First graph represents the diameter of each cluster: ****, p <0.0001. Second graph represents the number of clusters: ****, p <0.0001. The third graph represents the area of each cluster: ****, p<0.0001. The fourth graph represent the circularity of clusters: **, p= 0.0064; ****, p<0.0001.)


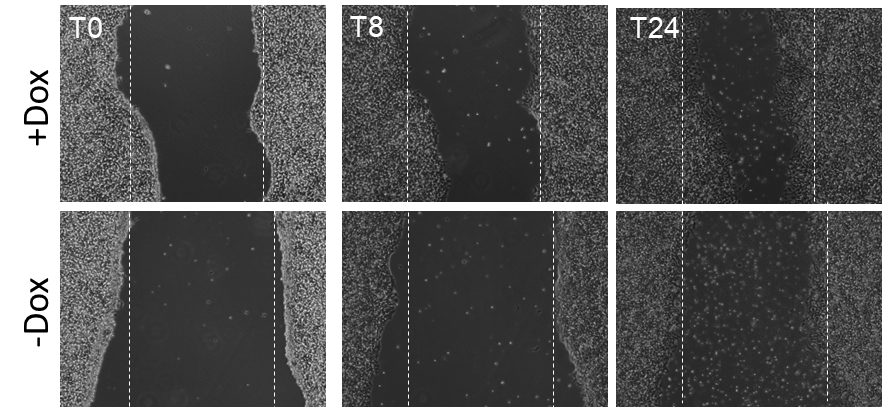


**Figure S4.** (2D migration assay (wound healing assay). A673/TR/shEF1 +/- DOX were seeded in a multiwell plate until reaching confluence. Images of the scratch are reported at 0. (T0), 8 (T8) and 24 (T24) hours.)


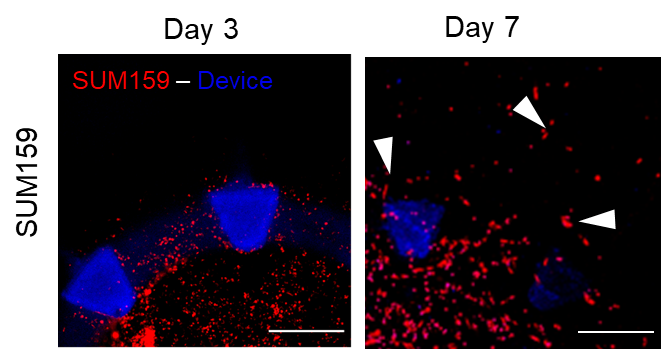


**Figure S5.** (Positive control for single cell migration assay, with highly aggressive SUM159 breast cancer cells seeded in the inner chamber and empty fibrin gel in the middle chamber, showing massive migration after 7 days.)


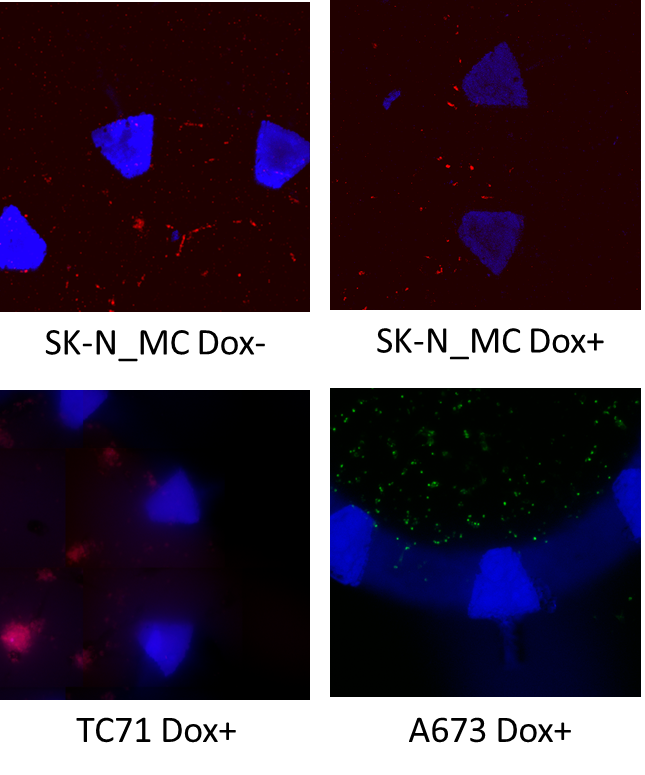


**Figure S6.** (Migration of different EwS cell lines, both with high (Dox-) and low (Dox+) expression of EWS::FLI-1, in the device with fibrin only in the middle chamber (B) after 21 days of culture. No migration of tumor cells has been detected.)


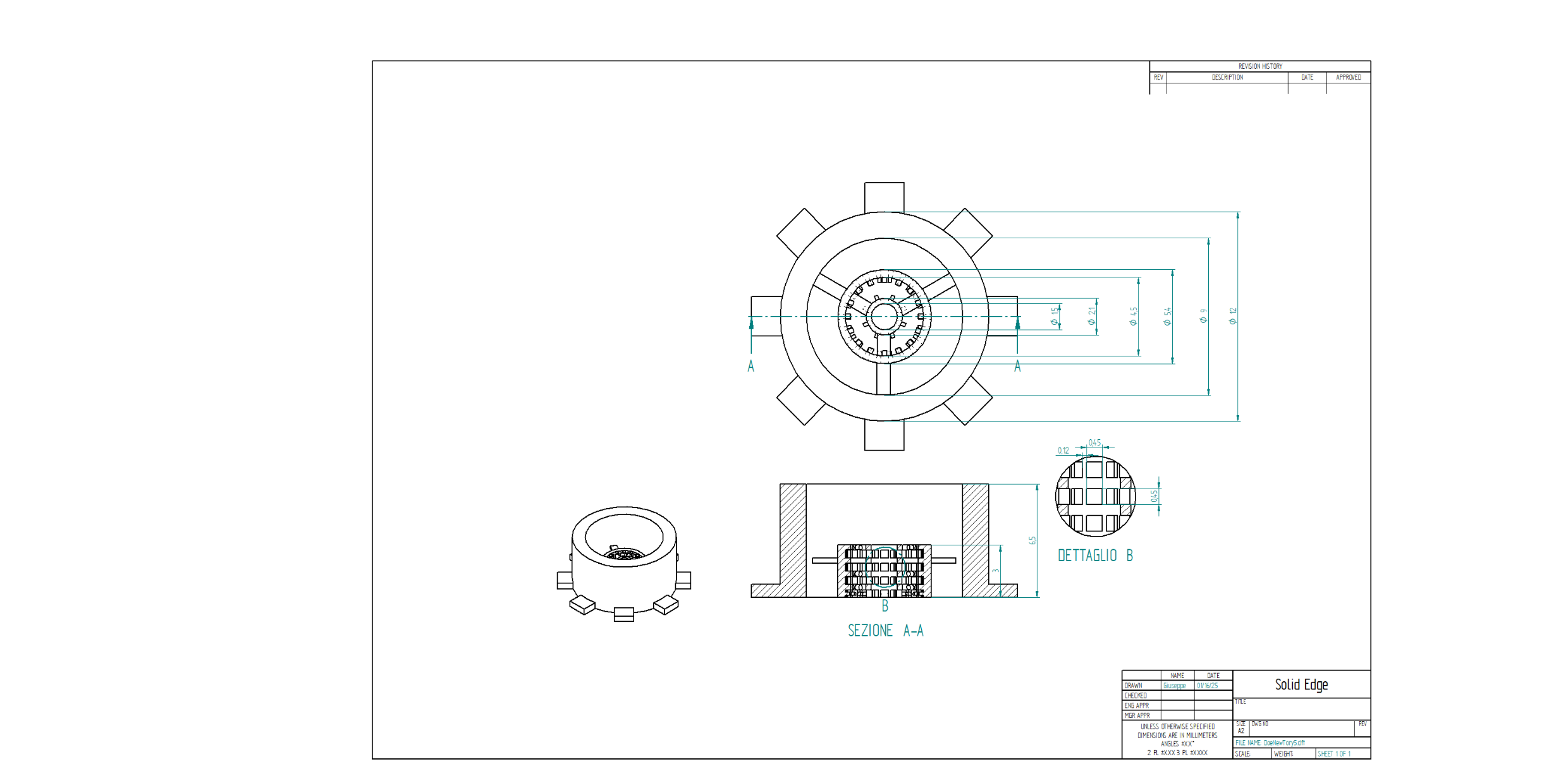


SECTION A_A

DETAIL B

**Figure S7.** (CAD drawing of the device, with all the relevant measures)
